# The Statistics of Eye Movements and Binocular Disparities during VR Gaming: Implications for Headset Design

**DOI:** 10.1145/3549529

**Published:** 2023-01-19

**Authors:** AVI M. AIZENMAN, GEORGE A. KOULIERIS, AGOSTINO GIBALDI, VIBHOR SEHGAL, DENNIS M. LEVI, MARTIN S. BANKS

**Affiliations:** University of California, Berkeley, USA; University of California, Berkeley, USA; University of California, Berkeley, USA; University of California, Berkeley, USA; University of California, Berkeley, USA; University of California, Berkeley, USA

**Keywords:** Computing methodologies → Perception, Virtual reality, Human-centered computing → Virtual reality, HMD, video games, stereopsis, eye movements, binocular disparity, eye tracking, vergence-accommodation conflict, virtual reality, field of view

## Abstract

The human visual system evolved in environments with statistical regularities. Binocular vision is adapted to these such that depth perception and eye movements are more precise, faster, and performed comfortably in environments consistent with the regularities. We measured the statistics of eye movements and binocular disparities in virtual-reality (VR) - gaming environments and found that they are quite different from those in the natural environment. Fixation distance and direction are more restricted in VR, and fixation distance is farther. The pattern of disparity across the visual field is less regular in VR and does not conform to a prominent property of naturally occurring disparities. From this we predict that double vision is more likely in VR than in the natural environment. We also determined the optimal screen distance to minimize discomfort due to the vergence-accommodation conflict, and the optimal nasal-temporal positioning of head-mounted display (HMD) screens to maximize binocular field of view. Finally, in a user study we investigated how VR content affects comfort and performance. Content that is more consistent with the statistics of the natural world yields less discomfort than content that is not. Furthermore, consistent content yields slightly better performance than inconsistent content.

## 1 INTRODUCTION

The natural environment is structured in ways that have a significant impact on visual experience. The environment contains many opaque surfaces that occlude the view of farther surfaces. It is also strongly influenced by gravity, so many surfaces are earth-horizontal (e.g., grounds, floors, table tops) or earth-vertical (trees, walls). Furthermore, people do not fixate random points in the world, but rather behaviorally significant points. These environmental and behavioral properties lead to statistical regularities in the images formed on the retinas.

**Fig. 1.**
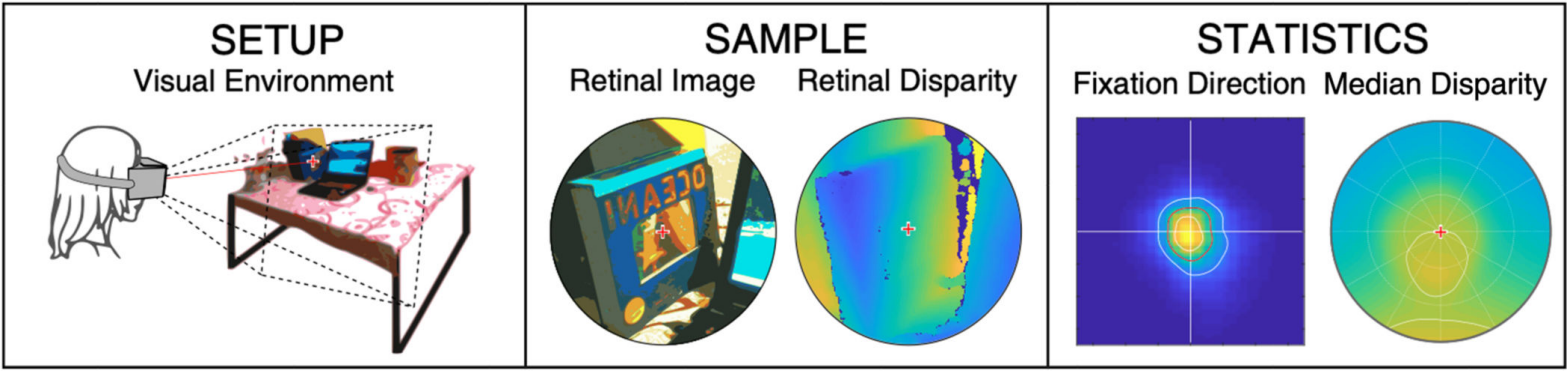
Eye movements and binocular disparities in VR-gaming environments. We measured binocular eye movements and retinal disparities as people played video games in a virtual (HMD) environment. The left panel depicts the situation. The central panel shows example images seen in the environment and the corresponding retinal disparities. The red crosses are the point of fixation. The right panel shows statistics of fixation directions and retinal disparity.

The human binocular visual system is adapted to these naturally occurring regularities. As a result, depth perception and eye movements in the real world are generally fast, precise, and performed with comfort. Virtual environments, such as virtual-reality (VR) games in head-mounted displays (HMDs), may or may not be compatible with the regularities to which the visual system has become adapted. Incompatibility could well cause viewer discomfort and reduced visual performance. A major purpose of the work presented here is to measure the statistics of fixations and binocular disparity in VR-gaming environments (Figure 1) in order to assess the compatibility of those statistics with adaptations made by the visual system, and to evaluate the degree to which current headsets and games are compatible with those statistics. For a summary, please watch the supplemental video.

Our key contributions are as follows:

- *Statistics of eye fixations in the VR-gaming environment*. We measured the directions and distances people fixate. The distributions of fixation directions and distances are more restricted in VR gaming than in the natural environment.
- *Statistics of binocular disparity in the VR-gaming environment*. We measured the distribution of disparity across the visual field when people play popular video games. From previous work we know that the distribution from the natural environment has a consistent tendency for near disparities below fixation and far disparities above. That tendency is less prominent and regular in the VR environment. Experiencing double vision is more likely in VR.
- *Discomfort and performance with consistent and inconsistent stimuli*. We conducted a user study in which we presented scenes that were consistent or inconsistent with natural statistics. We found that discomfort was greater and performance poorer with inconsistent scenes.
- *Probability of vergence-accommodation conflicts in the VR-gaming environment*. From the distribution of fixation distances, we determined how likely it is for conflicts to occur that are large enough to cause viewer discomfort. We calculated the screen distance that minimizes the probability of large conflicts.
- *Screen positioning that maximizes the binocular field of view in the VR-gaming environment*. Given the distance people tend to fixate, we found that the optimal placement of screens is slightly nasal, which differs from the more common temporal placement.

## 2 BACKGROUND

Having two eyes to view the world is both advantageous and challenging. The advantage is that the differences in the two views—binocular disparities—can be used to precisely compute the 3D layout of the visible environment. The challenge is the difficulty of solving binocular correspondence: Which point in one eye’s image arose from the same place in the scene as a point in the other eye’s image? Imagine solving binocular correspondence in an environment consisting of small objects randomly distributed in three space (as described by Sprague et al. [2015]). In every direction, all distances would be equally probable, so disparities would have a very broad distribution. Accordingly, the search for correspondence solutions would have to encompass an especially large range of disparities.

But the natural environment is very different from this. It contains many occluding surfaces and many earth-horizontal and earth-vertical surfaces. And viewers do not fixate randomly, but rather fixate behaviorally significant points such as surfaces upon which they are walking and objects they are manipulating [Land et al. 1999; Matthis et al. 2018]. They also generally view the world with the head upright. These environmental and oculomotor constraints are evidenced by the brain’s search for solutions to binocular correspondence: They allow a much more restricted and efficient search than would otherwise be required [Sprague et al. 2015]. In fact, the human visual system has adapted to these constraints such that it functions best (faster, more accurately, and with greater comfort) in environments that are similar to the natural environment. A major goal of the work presented here is to determine the degree to which the disparities experienced in VR conform to those of the natural environment.

Another important aspect of visual function is the coordination of binocular eye movements and the focusing response of the eyes: i.e., vergence (converging or diverging the eyes to be aligned on the object of interest) and accommodation (changing the power of the eye lens to focus the object of interest). These responses are neurally coupled. As a consequence, converging (or diverging) the eyes causes the eye lens to increase (or decrease) power. And accommodating by increasing (or decreasing) the lens power causes the eyes to converge (or diverge) [Fincham and Walton 1957; Schor 1992]. Stereoscopic displays, including HMDs, require the visual system to uncouple these responses because the viewer may have to converge or diverge to fuse an object in front of or behind the screen while maintaining accommodation at the screen distance [Kooi and Toet 2004]. This *vergence-accommodation conflict* is known to cause a variety of user issues including discomfort, reduced performance, and distortions of 3D percepts [Akeley et al. 2004; Häkkinen et al. 2006; Hoffman et al. 2008; Koulieris et al. 2017; Lambooij et al. 2009; Mauderer et al. 2014; Shibata et al. 2011; Urvoy et al. 2013; Watt et al. 2005]. An important goal of the work reported here is to determine the statistics of vergence-accommodation conflicts in VR gaming in order to provide guidelines for minimizing the conflict.

When people make upward, leftward, and rightward saccades, they tend to diverge the eyes. When they make downward saccades, they tend to converge [Collewijn et al. 1988; Enright 1984; Gibaldi and Banks 2019]. These biases in saccadic-related vergence are consistent with the statistics of the natural environment and thereby enable the oculomotor system to make accurate movements in the real world. Another goal of our work is to determine whether or not the statistics of virtual scenes conform to natural statistics and, if they do not, to make recommendations on how to modify the statistics to aid oculomotor behavior.

The screens in HMDs have wider temporal fields (toward the ears) than nasal fields (toward the nose). This increases the total field of view (the regions seen by one or the other eye), but decreases the binocular field of view (the regions that are imaged on corresponding regions in the two eyes). Another goal of the work presented here is to use the statistics of fixation distances to determine the screen placements that would maximize the binocular field of view.

## 3. RELATED WORK

### 3.1 Eye Movements in the Natural Environment

Researchers have measured the eye movements people make when performing everyday tasks in the natural environment. The overarching result is that people fixate behaviorally significant points in the scene and that that tendency depends on the task being performed.

Land and colleagues [1999] measured where people fixate when performing a familiar task: Making a cup of tea. They found that nearly all fixations were either on the object currently being manipulated or on one soon to be manipulated.

Matthis and colleagues [2018] measured fixations as people walked on rugged or flat terrain. They found that nearly all fixations were on places in the path where the person will soon be placing the feet, and that fixations were farther ahead on flat than on uneven terrain.

Other researchers have measured the statistics of fixation directions as people engage in a variety of everyday tasks [Kothari et al. 2020; Sprague et al. 2015; Tatler and Vincent 2008]. They found that most directions fall within ±15° of straight ahead. They also found that horizontal deviations from straight ahead are more common than vertical, that downward deviations are more common than upward, and that horizontal and vertical deviations are more common than oblique.

### 3.2 Eye and Head Movements in HMDs

Researchers have investigated eye and head movements when people use HMDs. Some have compared those movements in the HMD environment to those in natural viewing.

Kollenberg and colleagues [2010] measured performance and eye movements while subjects performed a visual-search task in an HMD and in natural viewing. Subjects performed more poorly with the HMD (i.e., search time was greater) and made smaller and more frequent saccadic eye movements in the HMD. Pfeil and colleagues [2018] compared eye and head movements in an HMD and in natural viewing while subjects performed visual-search and reading tasks. They also included a restricted-field, natural-viewing condition in which subjects wore the HMD but with the display screen and optics removed. Their results showed that subjects were much more likely to make combined eye and head movements in the HMD than in natural viewing. Their modified HMD elicited behavior that was more similar to that in the natural environment than in the HMD environment. The researchers did not state the field of view in that condition, so it is difficult to know whether restricted field of view or some other HMD property produced the differences in behavior between HMDs and natural viewing.

Sidenmark and Gellersen [2019] measured eye, head, and body movements while people explored a virtual environment with an HMD. Subjects rarely made eye movements more than ±10° left or right of straight ahead in head coordinates and tended to move their heads frequently as they explored the environment. Sitzmann and colleagues [2018] measured eye and head movements while people explored virtual environments with an HMD or a desktop display. They observed a clear tendency for gaze direction to center around the horizontal midline in both environments probably because the horizon was a prominent feature in the display content. This is consistent with the finding that subjects rarely make eye movements more than ±10° from straight ahead, and tend to move their heads frequently as they explore the environment.

### 3.3 Vergence-Accommodation Conflict

The vergence-accommodation conflict and its effect on viewer comfort, performance, and perception have been extensively reviewed [Koulieris et al. 2019; Kramida 2015; Lambooij et al. 2009; Urvoy et al. 2013]. Several researchers have documented its adverse effect on comfort [Hoffman et al. 2008; Koulieris et al. 2017; Padmanaban et al. 2017; Shibata et al. 2011], performance [Akeley et al. 2004; Hoffman et al. 2008], and 3D percepts [Mauderer et al. 2014; Watt et al. 2005]. This has led to novel, near-eye displays that minimize the vergence-accommodation conflict [Dunn et al. 2017; Hu and Hua 2014; Hua and Javidi 2014; Johnson et al. 2016; Konrad et al. 2017; Matsuda et al. 2017; Padmanaban et al. 2017; Rathinavel et al. 2019; Ueno and Takaki 2018; Yoo et al. 2020]. This is a very active area of research that is yielding ever better solutions to the problem.

## 4 METHODS FOR MEASURING FIXATION AND DISPARITY STATISTICS

We measured the distributions of gaze direction and distance, and the distribution of binocular disparity across the visual field during video-game play in an HMD. Unfortunately, video-game companies did not allow access to the 3D structure of virtual scenes during game play.^1^ To circumvent this issue, we developed four games in Unity (version 2019.3.8f1), and saved gaze data and depth buffers during game play. The four games were designed to be representative of popular VR video games (Section 4.4).

### 4.1 Depth Buffer Acquisition

To save the 3D geometry of the environment during game play, we acquired the scene depth using Render Textures in Unity. At runtime, a depth render texture is created where each pixel value of the texture contains a high-precision depth value. The value represents Unity view-space depth ranging non-linearly between [0,1] with a precision of 16 bits, depending on the platform and game configuration. We converted from buffer values to distances in meters.

Textures were acquired for each game for the left eye at a minimum of 40 depth frames per second. Saving these textures to disk during runtime can affect game play by reducing frame rate. To ensure the best user experience, we down-sampled the textures by a factor of 4, encoding them to 363 × 403 PNG images before saving to disk. We found that this resolution was more than adequate for measuring fixation and disparity statistics. Examples of the depth buffers for each game.

### 4.2 Apparatus

**Fig. 2.**
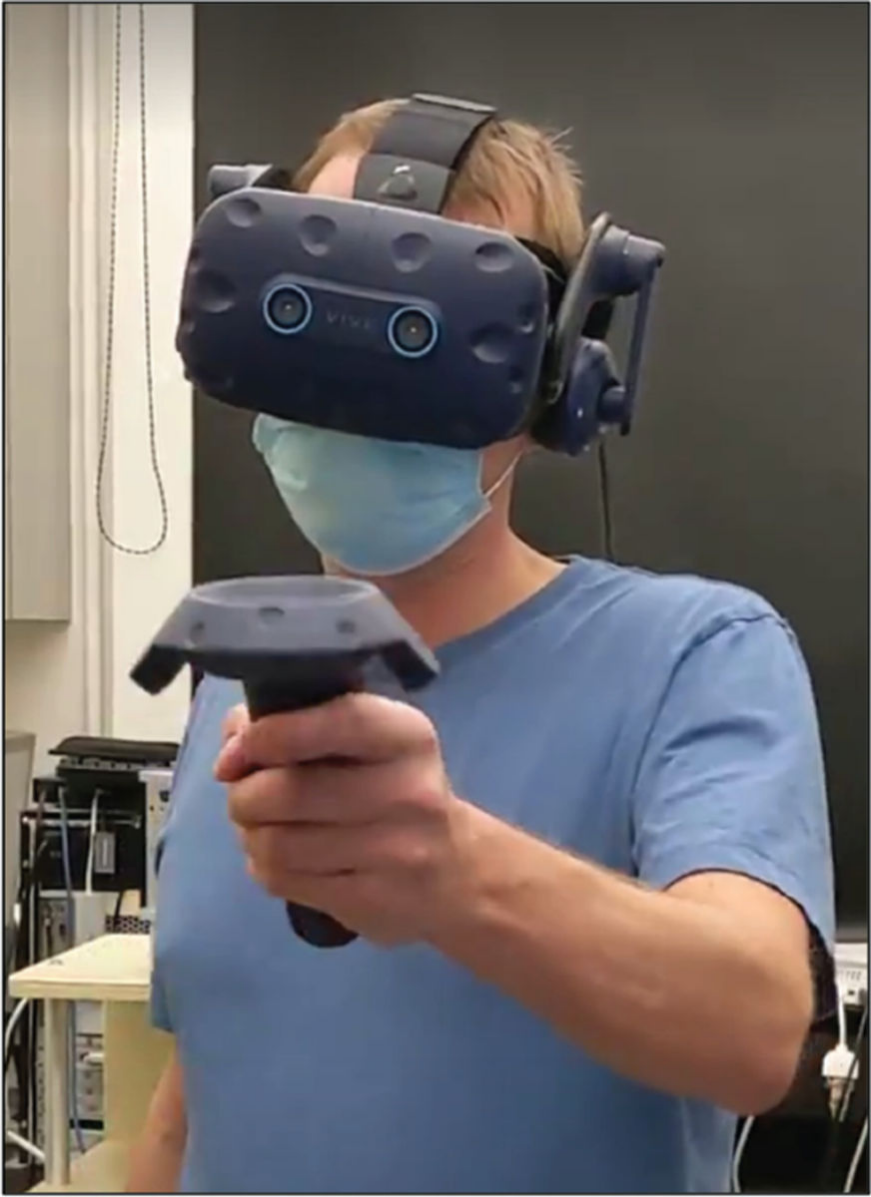
The headset and controller used as subjects played video games. The headset is an HTC Vive Pro Eye.

Video games were presented using the HTC Vive Pro Eye headset shown in Figure 2, which includes a built-in eye tracker (Tobii XR). The Tobii XR SDK V1.8.3 [Tobii 2020] and Vive SRanipal SDK V1.1.0.1 [Vive 2020b] were used to access tracking data at 90 Hz. According to the manufacturer, tracking accuracy is ~0.5–1.1° [Vive 2020a]. The HMD includes two OLED screens, one for each eye, with a resolution of 1400 × 1600 pixels per eye.

**Fig. 3.**
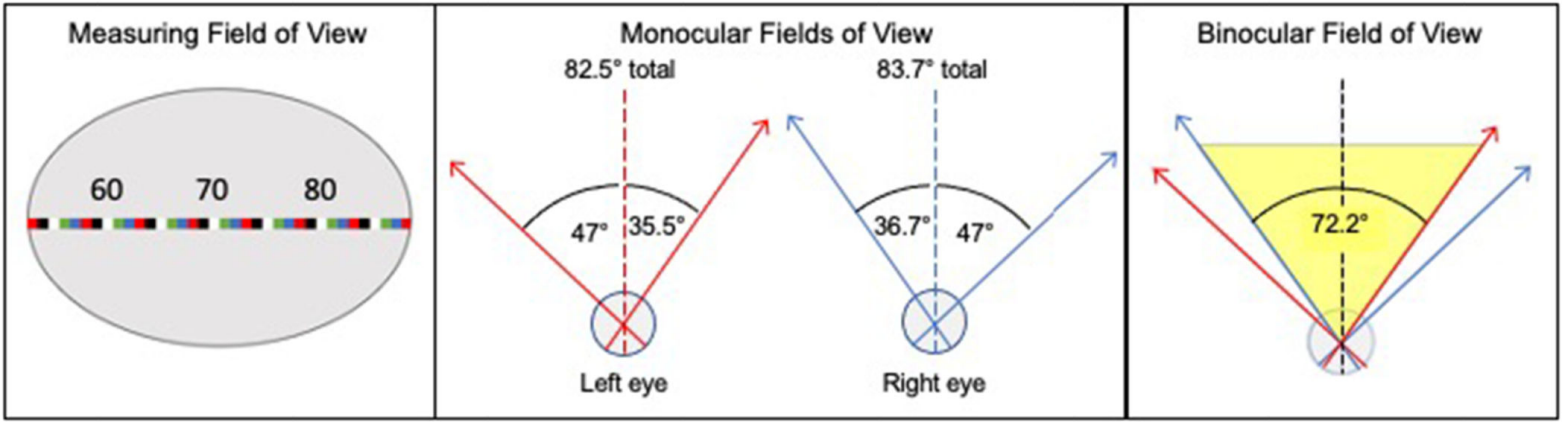
Horizontal field of view in the headset. (Left) 2 × 2 cm cubes presented at a virtual distance of 100 cm. Every 10th cube is numbered as shown. Participants indicated the leftmost and rightmost cubes they could see with the left and right eyes. The same procedure was used to determine the vertical field of view with the cubes in a vertical stack. (Middle) Horizontal visual fields for the left and right eyes. (Right) Horizontal binocular field of view in yellow.

We measured the monocular and binocular fields of view in the Vive Pro Eye. To do this, we generated a row or column of colored cubes each 2 cm wide and high in the virtual scene at a distance of 100 cm (Figure 3, left panel). Two of the authors wore the headset and viewed the cubes with just the left eye or just the right. To assess the horizontal field of view, they indicated the leftmost and rightmost cubes that were visible to the left and right eyes. They did the same for the highest and lowest visible cubes. The results differed slightly from one author to another because the distance from their eyes to the screen differed. From the average measurements, we determined that the monocular fields extend ~47° from straight ahead temporally (i.e., left limit for left eye and right limit for right eye) and ~36° nasally (right and left limits for left and right eyes, respectively). They extend ~93° vertically in both eyes. Thus, the monocular fields are each ~83° horizontally and ~93° vertically (Figure 3, middle panel). Consequently, with the eyes in forward and parallel gaze (i.e., vergence = 0°), the binocular field is ~72° wide and ~93° high (Figure 3, right panel). These values agree reasonably well with previous reports [Vive 2020a].

According to the manufacturer, the optical distance from the viewer’s eye to the screen is 65 cm (1.54 diopters). We made our own measurements of this distance. We used a camera with short depth of field positioned where the eye is meant to be. We focused the camera on the displayed content and then, without changing focus, moved the camera to an optical bench where we translated it relative to an eye chart to find the best focus distance. We obtained the same result as was reported by the manufacturer.

The games were run on a PC with a Windows 10 64-bit operating system, an Intel(R) core(TM) i7–8700k processor with 3.7 GHz, 48 GB RAM, and two NVIDIA TITAN V graphics cards. The video-game frame rate reached ~80 Hz.

### 4.3 Participants

Ten people with normal or corrected-to-normal visual acuity (20/32 or better in the Bailey-Lovie test [Bailey and Lovie 1980]) and normal stereo acuity (30 arcsec or better in the Randot stereo test [Okuda et al. 1977]). They were 23–37 years of age. The experimental protocol was approved by the Institutional Review Board at our university in accordance with the Declaration of Helsinki. Participants signed informed consent forms before participating.

### 4.4 Video Games

Participants each played four video games for 3 minutes each. The order of game presentation was counterbalanced using a Latin Square design. Our games were designed to be representative of the most popular VR games. We used data from Steam [2020], the video-game distribution platform, to guide our game designs. The selected games have a representative range of depths (far, middle, and near/reach space) and tasks (first-person shooter, rhythm game, environment simulation). The games were the following:

- *Rhythm Game (mid/near depth task)*: Cubes representing the beats of background music move toward the player. The player swipes at the cubes with a saber. This game is similar to *Beat Saber*^*®*^, the 3rd most popular VR game [Steam 2020].
- *First-Person Shooter Game (near/mid/far depth task)*: Zombies in a haunted graveyard approach the player. Players kill them using a gun and axe. This game is similar to *Arizona Sunshine*^*®*^, the 4th most popular VR game [Steam 2020].
- *Environmental Simulation Game (near depth task)*: To escape a cabin, players must complete tasks that are revealed as they explore the cabin. This game is most similar to *Job Simulator*^*®*^, the 21st most popular VR game [Steam 2020].
- *Action-Rhythm First-Person Shooter Game (mid/far depth task)*: Players are transported forward along a path. Enemies appear randomly and shoot at the player who must shoot the enemies or dodge the bullets to avoid being hit. This game is most similar to *Pistol Whip*^*®*^, the 17th most popular VR game [Steam 2020].

Example frames from the games are provided in Supplementary Figure S1.

### 4.5 Calibration and Validation

At the beginning of each session, the participant placed and adjusted the HMD on the head to a comfortable position that enabled a full field of view. They also adjusted the separation between the left and right screens to match the inter-ocular distance.

We then calibrated the eye tracker using the five-point calibration procedure provided by the Vive Pro Eye. The resulting data were affected by a constant translation along the *x* and *y* axes. We took this translation into account in post-processing. Slippage of the HMD on the participant’s head can invalidate the calibration. To check whether slippage had occurred during an experimental run, we developed our own procedure to enable more accurate and consistent tracking. A small target was displayed at different positions in the central visual field, and the participant was instructed to fixate its center and press a button once he/she thought fixation was accurate. The targets were displayed at virtual distances of 1.5 and 10 m. They were shown in random order in five positions at each distance; those positions were straight ahead and at eccentric points in a 2 × 2 matrix. The corner targets were 10° from the central target; we chose that range because it incorporates most of the gaze directions that occur in natural viewing [Sprague et al. 2015]. The procedure was performed before and after each game play. To assess tracking accuracy before testing began, we computed the RMS error between the known calibration points and the gaze directions indicated by the tracker and our algorithm. Sessions in which RMS exceeded 0.8° were discarded (which occurred about 1/3 of the time). We chose 0.8° as the criterion because that value is similar to the repeatability of the eye tracker. We performed the calibration again after each game play to determine if slippage of the headset had occurred. We required that the RMS error between pre-test and post-test was less than 1.0°. If this criterion was exceeded, the participant repeated the whole session: pre-calibration, game play, and post-calibration. Each participant contributed a full set of data for each of the four video games even if it required repeating one or two of the games.

### 4.6 Post-processing

Gaze direction for both eyes and retinal disparity were computed in post-processing.

The data from the eye tracker were used to compute the pixel position of the fixation point for each eye in the left depth buffer image, and their binocular combination. In order to collect statistics of natural retinal disparity, we included all gaze samples in which the eyes were either stationary or moving slowly enough for the visual system to process disparity. The slow movements are smooth pursuit, vergence, and the vestibulo-ocular response. Gaze samples recorded during a saccade were not included because saccadic suppression and motion smearing prevents disparity processing. To identify samples during saccades we defined a saccade as movements exceeding a velocity of 60°/s. The start and end points of the saccade were defined as, respectively, 2% and 98% of the saccadic amplitude [Gibaldi and Banks 2019; Gibaldi and Sabatini 2021]. The depth buffer and eye position returned by the eye tracker were used to transform the screen-referenced gaze data into real-world, cyclopean-eye–referenced coordinates, using the screen center to set the reference azimuth and elevation for the estimated binocular gaze directions.

**Fig. 4.**
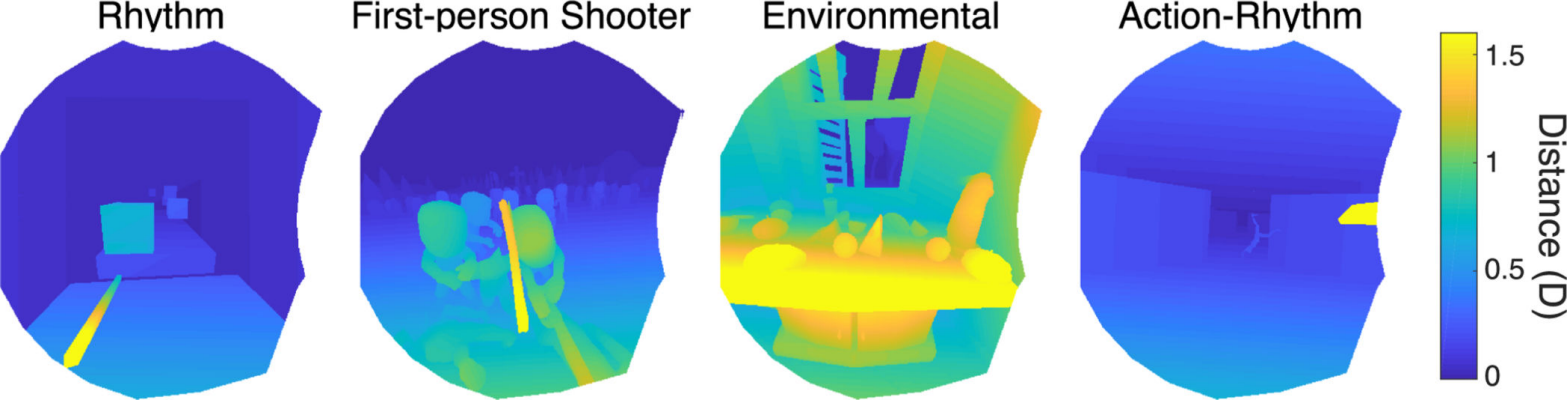
Example depth-buffer values from the video games. One frame seen by the left eye is shown from left to right for the Rhythm, First-person Shooter, Environmental, and Action-Rhythm games. Colors represent distance in diopters as indicated by the color bar.

The depth buffers shown in Figure 4, were used to reconstruct the 3D scene [Canessa et al. 2017]. The gaze data were then mapped into the reconstructed scene, and the 3D scene was projected into the left and right eyes to compute the retinal disparities experienced by the subject given where they were fixating [Gibaldi et al. 2017]. In natural binocular vision, not all points in the 3D scene are visible to both eyes, especially near depth discontinuities. Disparity is not defined for such regions so those regions were of course not included in our statistics. We also incorporated expected eye torsion in the analysis by employing Listing’s Extended Law (L2) with a gain of 0.8, which is the most common gain in people with normal binocular vision [Somani et al. 1998].

**Fig. 5.**
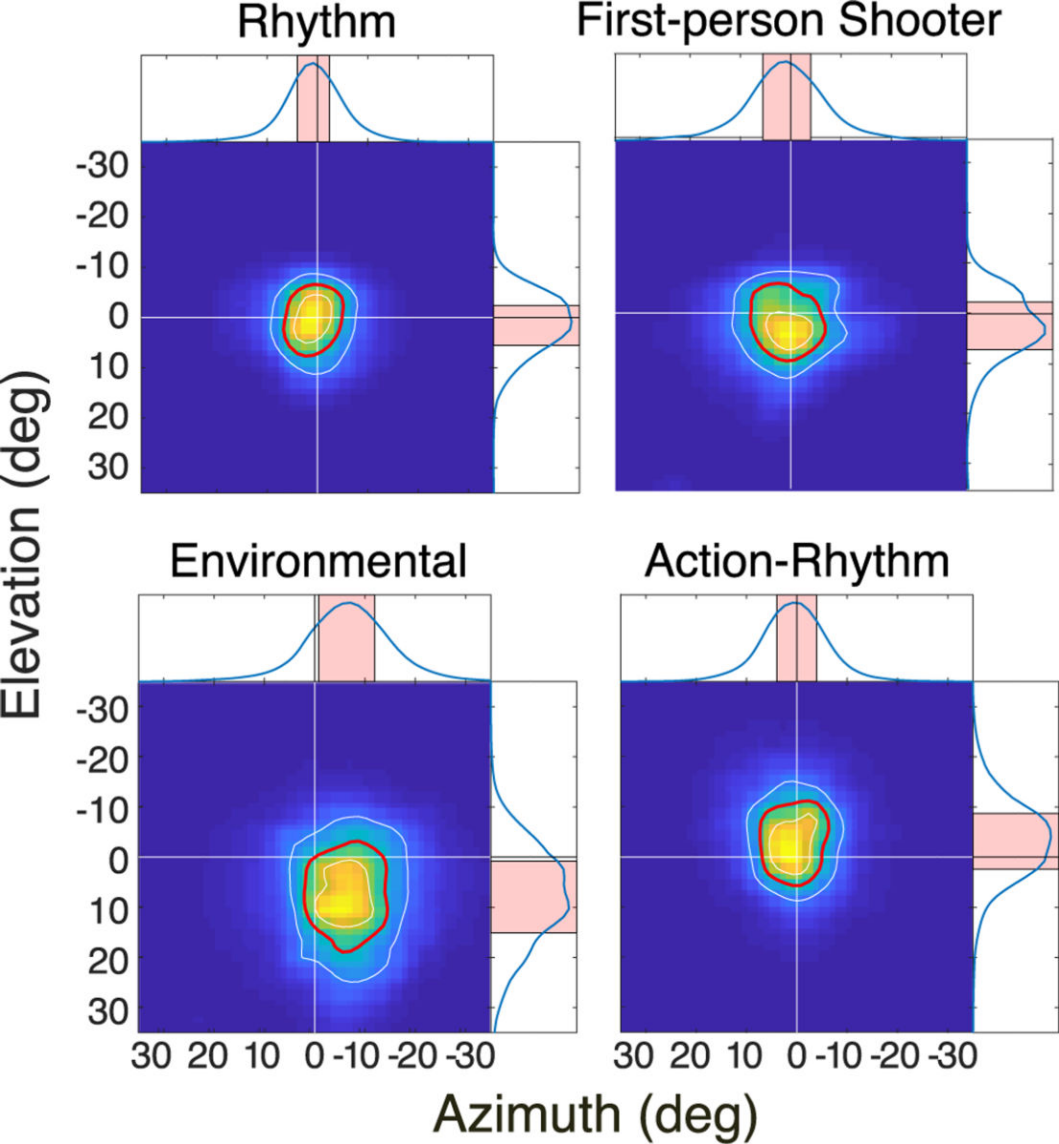
Probabilities of fixation directions in head coordinates. Individual panels plot the probability of fixation directions, averaged across subjects, for each game. Horizontal gaze direction is on the horizontal axis and vertical on the vertical axis. Red contours show the region containing 50% of fixations. White contours are 25th and 75th percentiles. Marginal probabilities are shown on the right and above. Pink areas represent 50% of the fixation directions.

**Fig. 6.**
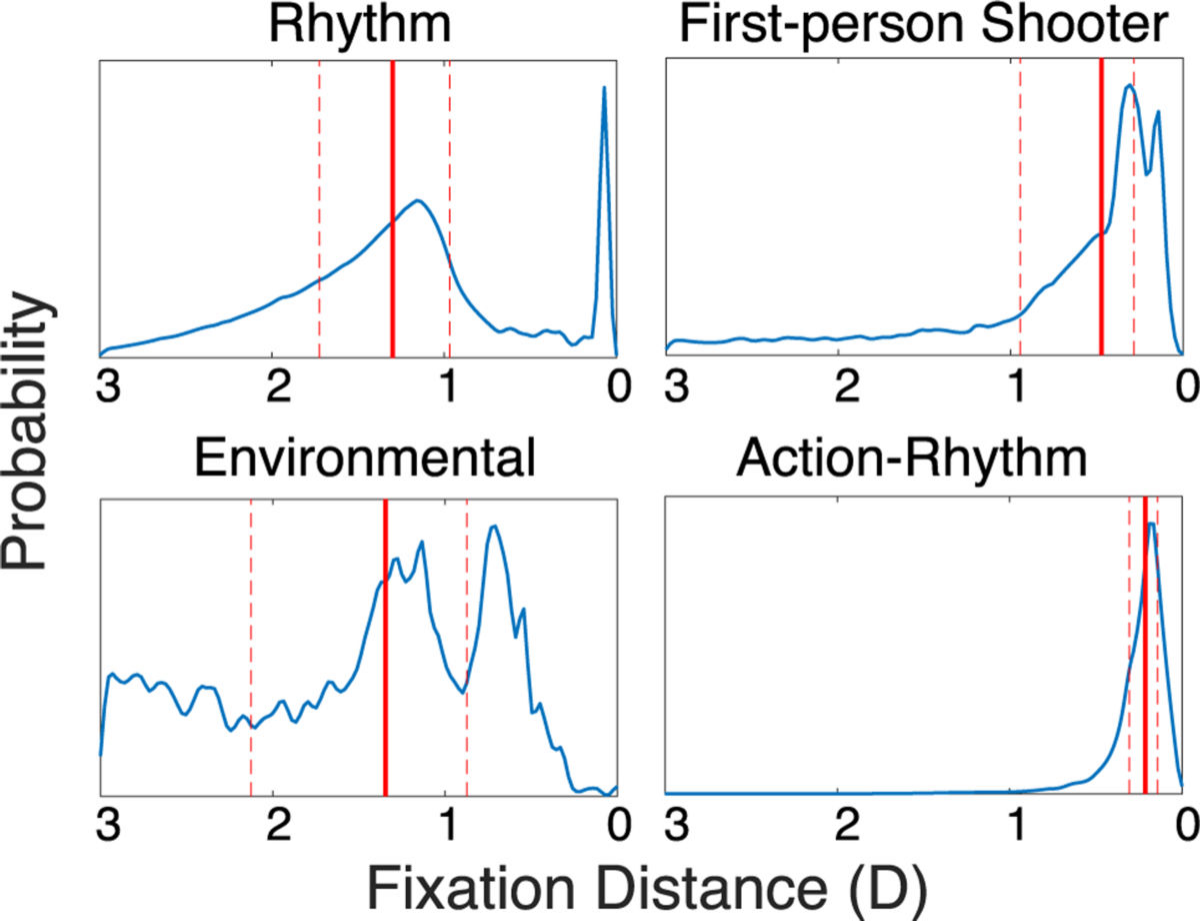
Probabilities of fixation distances. Individual panels plot the probability of fixation distances in diopters, averaged across subjects, for each game. Near distances are on the left in each panel and far ones on the right. Median fixation distances are represented by the solid red lines, and 25th and 75th percentiles by the red dashed lines.

For summary statistics, we combined the data across the four games giving equal weight to each game. This yielded average statistics for gaze direction and distance (Figures 5 and 6) and binocular disparity (Figure 9). Data from the four games, as well as summary statistics, are available at https://doi.org/10.6078/D1BB16.

### 4.7 Disparity Definitions

There can be some confusion about how to quantify binocular disparities. It is first of all important to make clear what coordinate system is being used. We use Helmholtz coordinates where azimuth is measured by latitude and elevation by longitude [Read et al. 2009].

It is also important to make a distinction between disparities relative to the viewer’s head and those relative to the viewer’s retinas. Head-centric disparities are unaffected by where the eyes are fixated, while retinal disparities are heavily influenced by fixation. The orientation of disparities is also commonly different in head and retinal coordinates. When referenced to the head, real scenes create many different values of Helmholtz horizontal disparities (i.e., differences in azimuth in the two eyes): the values depend on the distances of object points in the scene. Vertical disparities (i.e., differences in elevation in the two eyes) do not depend on scene geometry and are always zero [Read et al. 2009]. The goal in creating a stereoscopic display is to present the same disparities from the virtual scene as would be created by the analogous real scene. In such a display (when it is well-calibrated), horizontal disparities on the screens can take on many values, but vertical disparities are always zero. Said another way, object points are displayed on virtual horizontal lines, where the horizontal positions of the point for the two eyes can differ but the vertical positions cannot. Thus, headcentric disparities in the real world and in well-calibrated stereoscopic displays are oriented horizontally.

Disparities in retinal coordinates are heavily dependent on where the viewer is fixating. As a consequence, horizontal and vertical disparities can both take on non-zero values. They are both dependent on scene geometry, positions of object points relative to the head, and where the eyes are fixating. Retinal disparities in the real world and in well-calibrated displays, therefore, often have non-zero horizontal and vertical disparities, so they are generally oriented differently in retinal than in head coordinates. We mention this because the presence of non-zero vertical disparities creates a demand to make vertical vergence eye movements (i.e., one eye rotating up or down more than the other [Schor et al. 1994]) and this can cause discomfort [Kane et al. 2012]. With HMDs, this demand is not necessarily due to miscalibration; it can also be due to the contents of the virtual scene.

Additional methodological details are provided in the Supplementary Material.

## 5 RESULTS FOR FIXATIONS AND DISPARITIES

### 5.1 Fixation Directions and Distances

Figure 5 shows the distributions of gaze directions relative to the head for the four video games. The distributions from one game to the next are quite similar. They are narrow and nearly isotropic because there were few fixations that deviated more than 5° from straight ahead. The narrow distribution of fixation directions in HMDs has been reported by others who have hypothesized, as we do, that people tend to make small eye movements and large head movements due to the restricted field of view in HMDs compared to natural viewing [Pfeil et al. 2018; Sidenmark and Gellersen 2019; Sitzmann et al. 2018] (see Section 8.1). Additionally, the Vive Pro Eye HMD uses Fresnel lenses, characterized by an unsmooth grooved surface. Such lenses yield poorer optical quality in the periphery than in the center of the display. Thus, to maximize image quality near the fovea, participants may have turned the head rather than the eyes to avoid fixating regions of poor quality.

The fact that fixation directions are concentrated near straight ahead in the VR-gaming environment is useful information for foveated rendering applied to video games [Albert et al. 2017; Guenter et al. 2012; Patney et al. 2016]. Specifically, one might achieve more compute-time benefit than achieved with rendering coupled with eye tracking by not doing eye tracking and simply expanding the sharply rendered region to cover the great majority of fixation directions: roughly the central 10° (diameter).

Figure 6 shows the distributions of fixation distances for the four games. There are many distant fixations in all but the *Environmental* game. The modes of the distributions in the *Rhythm*, *First-person Shooter*, and *Action-Rhythm* games are close to 0 diopters D, which corresponds to distant gaze for which the eyes’ visual axes are parallel or nearly so. We examine the consequences of the tendency to fixate far in Section 5.2.

When a person looks at a near object off to the left or right, the object is closer to one eye than the other creating a larger retinal image in the closer eye. When the object is also up or down, the person must make a vertical vergence movement to fixate the object accurately [Schor et al. 1994] and this can produce discomfort [Kane et al. 2012] (Section 4.6). Figures 5 and 6 show that this combination of near gaze in an oblique direction is quite rare in the VR-gaming environment. Thus, the vertical disparities experienced in that environment are generally quite small and probably not problematic.

**Fig. 7.**
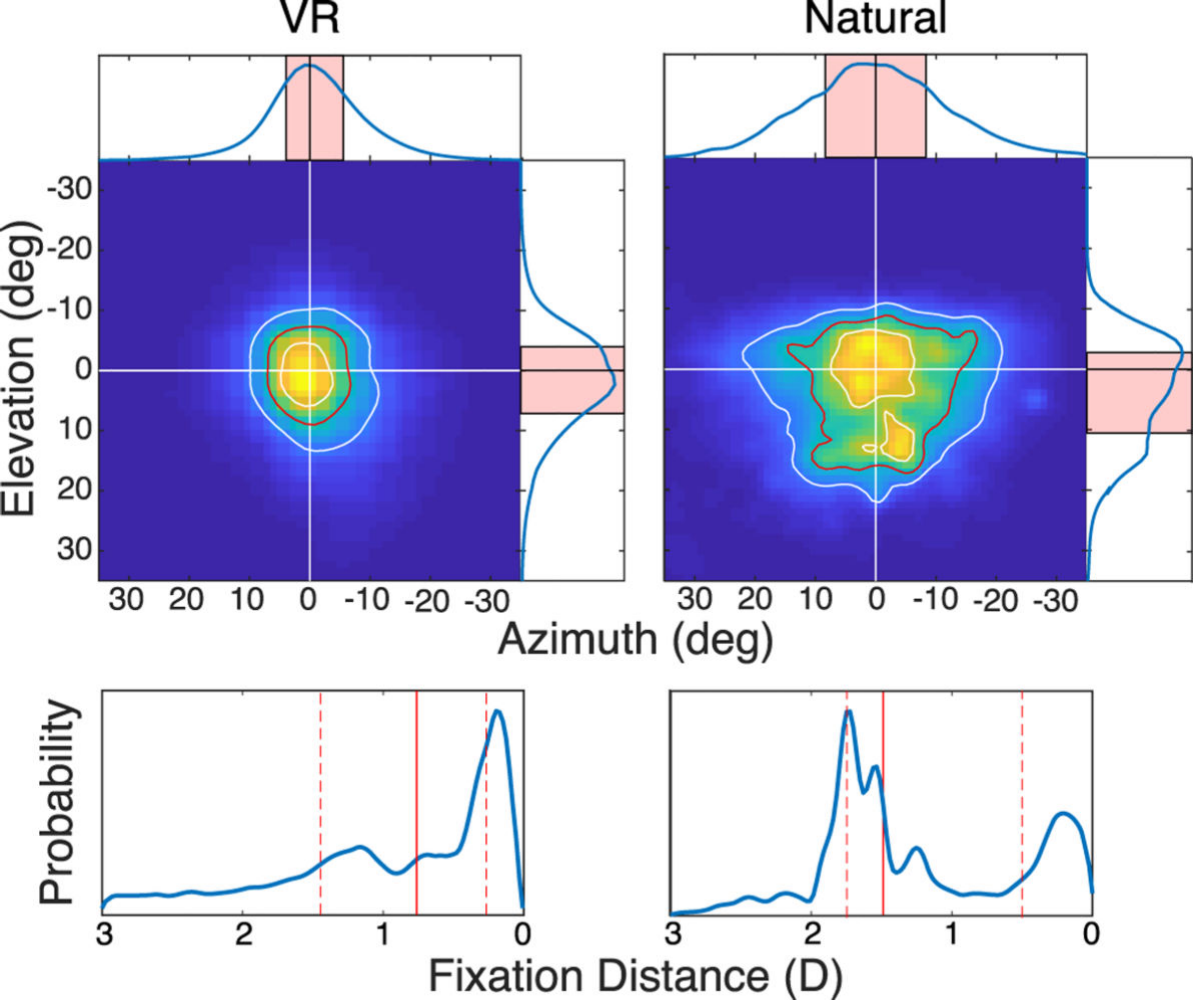
Probabilities of fixation directions and distances for VR and natural environments in head coordinates. The upper row plots the distributions of fixation directions. The VR distribution has been averaged across the four video games and the 10 subjects. The natural distribution has been combined in weighted fashion across six everyday tasks and the four subjects. Red contours show the regions containing 50% of fixations. White contours are 25th and 75th percentiles. Marginal probabilities are shown on the right and above. Pink areas represent 50% of the fixation directions. The lower row plots the distributions of fixation distances in diopters. Near distances are on the left and far on the right in each panel. Median fixation distances are represented by the solid red lines, and 25th and 75th percentiles by the red dashed lines.

Our main purpose in examining fixations in the VR environment is to determine how they compare to natural fixation behavior. Figure 7 enables the comparison by plotting both the VR data and data from natural viewing in the real world. The natural data were obtained from the BORIS dataset (https://github.com/Berkeley-BORIS) using methods described in Sprague et al. [2015] and Gibaldi and Banks [2019]. Those data are the weighted average across six everyday tasks and four subjects. The VR data are the average across the four games and 10 subjects.

The upper panels of Figure 7 plot the distributions of fixation directions from these averages. In the natural environment, the direction of gaze is most commonly straight ahead and slightly down relative to primary position. Secondary directions—leftward, rightward, upward, and downward—are the next most common [Gibaldi and Banks 2019; Kothari et al. 2020; Sprague et al. 2015; Tatler and Vincent 2008]. There are few gaze directions more than 15° from straight ahead because when people attempt to look at more eccentric points they usually execute a combined eye and head rotation [Barnes 1979; Guitton and Volle 1987; Pfeil et al. 2018]. The distribution of fixation distances in the VR environment is much narrower and more isotropic. The great majority of fixations is within 5° of straight ahead.

The lower panels of Figure 7 plot the distributions of fixation distances averaged across games and tasks. In the natural environment, we observe a broad distribution of distances with a median value of ~70 cm (1.5D); that distance is indicated by the solid red line. Of course, the distance of gaze varies significantly from one everyday task to another (Supplementary Figure S2). When walking outdoors, the most common distance is ~500 cm (0.2D). When making a sandwich, the most likely distance is ~62 cm (1.6D). The distribution of distances in the VR environment is generally farther than in the natural environment. The median VR value is ~125 (0.8D), which is indicated by the solid red line. The distances vary from one game to another (Figure 6), but are generally farther than in the natural environment. We consider the significance of this tendency to fixate far in Section 5.2.

### 5.2 Screen Distance and VA Conflict

Vergence and accommodation are negative-feedback control systems [Cumming and Judge 1986; Fincham and Walton 1957; Schor 1992]. The vergence part takes disparity as input and generates converging or diverging eye movements to null the disparity at the fovea. The accommodation part takes retinal blur as input and adjusts focus to minimize the blur. The vergence and accommodation parts of the control system work to drive their respective outputs to the same distance in the environment, so it makes sense that they communicate with one another through neural cross-links. Because of the cross-links, the act of converging or diverging causes the eye lens to change power (vergence accommodation) and the act of accommodating nearer or farther causes vergence movements (accommodative vergence). The cross-coupling increases speed and accuracy in the natural environment [Cumming and Judge 1986].

The cross-coupling is, however, counter-productive for viewing stereoscopic displays such as HMDs. In such displays, vergence must be to the distance of the virtual object of interest for a single, fused image to be seen. But the light comes from the display screen so accommodation must be to the screen distance for a sharp image to be seen. Thus, the distances for appropriate vergence and appropriate accommodation are often quite different. The difference is the *vergence-accommodation conflict*. When the conflict is nonzero, the visual system must work against the cross-coupling to fuse and sharpen the images. Larger conflicts cause greater deficits in perceptual performance, and considerable discomfort [Akeley et al. 2004; Hoffman et al. 2008; Koulieris et al. 2017; Mauderer et al. 2014; Shibata et al. 2011; Watt et al. 2005].

**Fig. 8.**
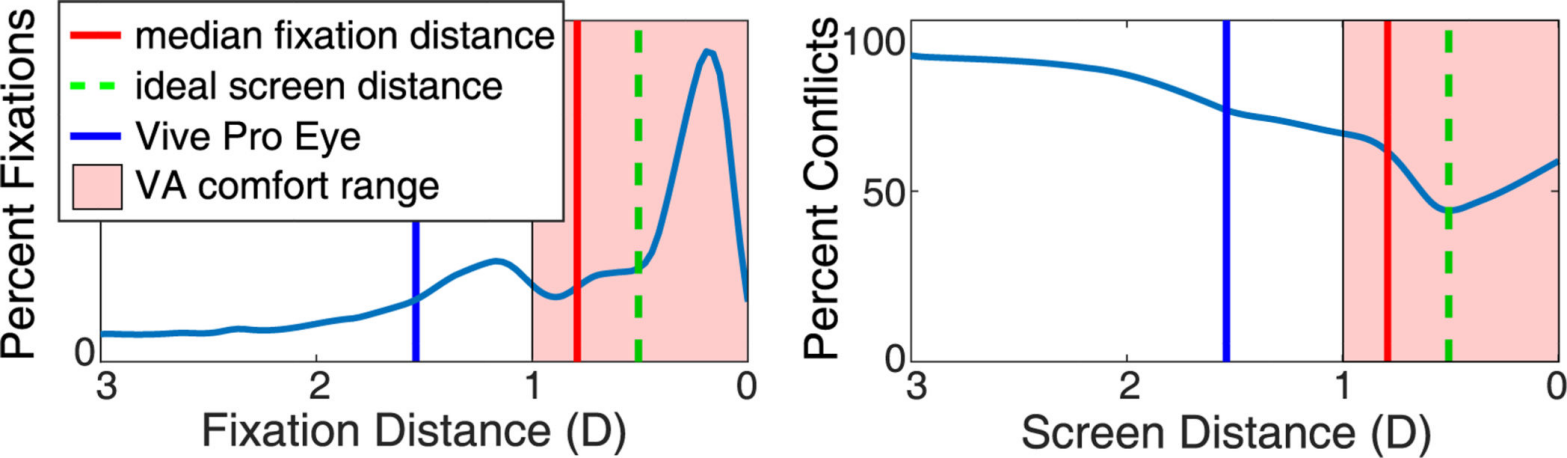
Probability of vergence-accommodation conflicts during video-game play. (Left) Percentage of fixations at various distances. The horizontal axis is the fixation distance in diopters and the vertical axis is the percentage of fixation distances averaged across the four games. These are the same data as in the lower left panel of Figure 7. Median fixation distance is indicated by the red solid line at 0.8D (125 cm). Optical distance of the screen in the HTC Vive Pro Eye is indicated by the solid blue line at 1.54D (65 cm). The pink patch represents a ±0.5D comfort range for the vergence-accommodation conflict, centered on the median fixation distance. (Right) Percentage of fixations generating uncomfortable vergence-accommodation conflict. The horizontal axis is screen distance in diopters and the vertical axis is the percentage of conflicts that exceed ±0.5D. The screen distance that minimizes the percentage of bothersome conflicts is indicated by the green dashed line at 0.51D (196 cm).

Current best practices in content development for HMDs recommend presenting virtual content at a distance similar to the optical distance of the screen in order to minimize discomfort due to the vergence-accommodation conflict [Oculus VR 2017]. We used our measurements of content and fixation statistics during game play to determine the distribution of the vergence-accommodation conflicts. Specifically, we used the distribution of fixation distances to determine how frequently those vergence distances would be nearer or farther than the optical distance of the screen by ±0.5D, thereby creating a conflict large enough to cause discomfort [Shibata et al. 2011]. Figure 8 shows the results. The left panel shows the percentage of fixations at various distances, averaged across the games and subjects; it is similar to the lower left panel of Figure 7. The median fixation distance is represented by the vertical red line. The right panel shows the percentage of fixations that are associated with conflicts greater than ±0.5D, as a function of screen distance. The screen distance in the Vive Pro Eye is indicated by the vertical blue line. The dashed green line represents the screen distance that would minimize conflicts. Obviously, it is much farther than the actual distance to the screen. Thus, discomfort due to vergence-accommodation conflicts would be reduced by nearly tripling the screen distance to 196 cm (0.51D). (The screen distances of other commercial devices (e.g., Oculus DK1, DK2, and CV1; HoloLens 1 and 2) are greater, but in most cases still not far enough to minimize conflict). Of course, the degree of mismatch will depend strongly on the specific demands of the virtual environment and task. Designers of HMDs and video games can use our data to better match screen and fixation distance to improve viewer comfort and performance [Koulieris et al. 2017].

### 5.3 Disparity Statistics

**Fig. 9.**
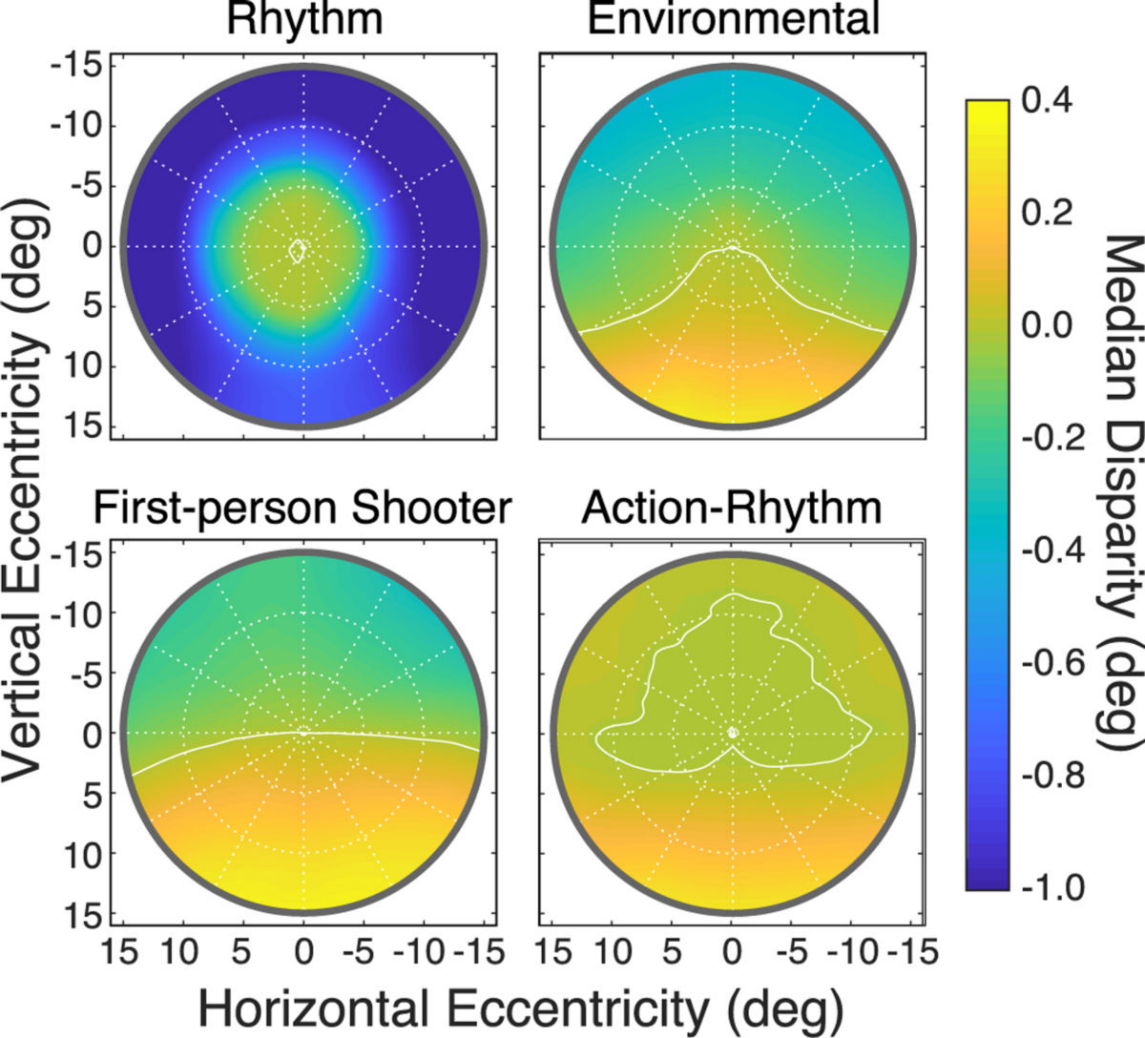
Median disparity and field position for the four video games. Each panel plots median horizontal disparity in retinal coordinates as a function of field position for one of the games, averaged across the 10 subjects. Fovea is in the middle. Upper visual field is up and left field is left. White contours represent zero disparity.

Figure 9 shows the median horizontal disparities at the retina for the four video games. As noted earlier (Section 4.6), the disparities are expressed in Helmholtz retinal coordinates. To determine disparities in those coordinates, we needed to know both the 3D scene geometry and where participants fixated in those scenes. The individual panels plot median disparity for each position in the visual field. Negative values (blue) correspond to uncrossed disparities (farther than fixation) and positive values (yellow) to crossed (nearer than fixation). In each panel, the fovea is in the center and the upper and left visual fields are at the top and left, respectively. The distributions vary across the four games. The *Environmental*, *First-Person Shooter*, and *Action-Rhythm* games generate a relatively small range of disparity with a trend from crossed in the lower field to uncrossed in the upper. The *Rhythm* game produced a much larger range with large uncrossed disparities a few degrees from the fixation point and no trend from crossed to uncrossed from the lower to the upper field. From these data it is clear, unsurprisingly, that the distribution of disparities across the visual field depends on the game being played.

**Fig. 10.**
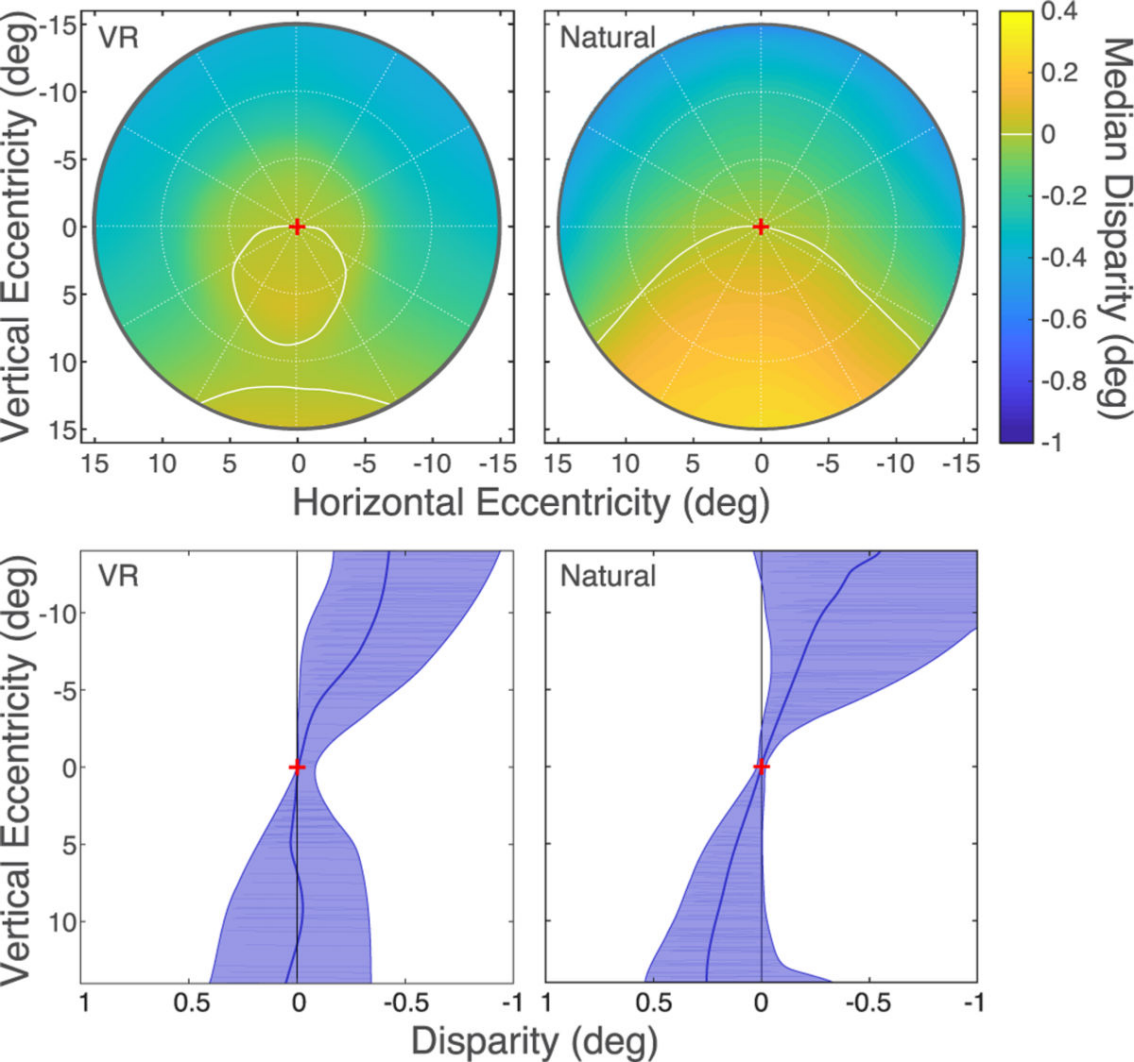
Median disparity as a function of field position for VR and natural environments. Upper panels: Median horizontal disparity in retinal coordinates for each field position. Fovea is in the middle. Upper visual field is up and left field is left. The white contours represent zero disparity. The left panel shows the data from the VR environment. The data have been generated by averaging across the four games and 10 subjects. The right panel shows the data from the natural environment. The data have been generated from the weighted average across six everyday tasks and four subjects. Lower panels: Cross sections along the vertical meridian. Disparity near the vertical meridian is plotted as a function of vertical eccentricity. Data for the VR and natural environments are in the left and right panels, respectively. The thick blue curves are the medians. Shaded areas indicate disparities between the 25th and 75th percentiles.

Our main purpose in measuring the disparities encountered in the VR environment is to determine how they compare to the disparities experienced in the natural environment. Figure 10 enables the comparison by plotting both the VR data and data from natural viewing in the real world. As stated earlier, the natural data were obtained from the BORIS dataset using methods described in Sprague et al. [2015] and Gibaldi and Banks [2019]. Those data are the weighted average across six everyday tasks and four subjects. The VR data are the average across the four games and 10 subjects. The right panels reveal clear regularities in naturally occurring disparities. The upper right panel shows median horizontal disparities across the visual field. There is a striking change from the lower to the upper field. The median disparity in the lower field is positive (crossed) while the median disparity in the upper field is negative (uncrossed). These are large tendencies. For example, 10° above fixation, 70% of disparities are negative. The top-back pitch of the data is highlighted in the lower right panel, which shows the median and range of disparity from the lower to the upper field. Thus, given where people tend to fixate, the natural environment creates a pattern of disparities that is slanted top back. The natural data also exhibit a systematic change from the left to the right field. Median disparity changes from negative (uncrossed) on the left to zero near the fovea to negative again on the right.

For humans to perceive depth from disparity, the visual system must determine which points in the left-eye’s image correspond to points in the right-eye’s image. The visual system utilizes the environmental regularities mentioned earlier to solve this binocular correspondence problem. Specifically, the search for disparity in a given location in the visual field is centered on corresponding retinal points. The definition of corresponding points is the following. For every retinal location in one eye there is a location in the other eye that forms a pairing with special status in binocular vision. These pairs are corresponding retinal points. Rays projected from those corresponding-point pairs intersect in the world on a surface called the *binocular horopter* [Ogle 1950; von Helmholtz 2013]. The horopter is pitched top back [Cooper et al. 2011; Nakayama 1977; Siderov et al. 1999]. So, for objects above current fixation to fall on the horopter they must be farther than fixation, while objects below fixation must be nearer. The horopter is also farther on the left and right (relative to the zero-disparity surface) than in the center.

Why is the horopter important? Binocular vision is best for objects on or near the horopter: fusion is guaranteed and depth discrimination is most precise [Blakemore 1970; Brewster 1844; Fischer 1924; Ogle 1950; Prince and Eagle 2000; Schumer and Julesz 1984; Vlaskamp et al. 2013]. Importantly, the shape of the horopter is quite similar to the central tendency of the natural-disparity statistics (Figure 10). Therefore, fusion and accurate stereopsis are guaranteed for the most likely natural scenes.

The disparity statistics are also relevant to oculomotor behavior. When people make upward saccadic eye movements to a stimulus whose distance is ambiguous, their eyes diverge and when they make downward saccades their eyes converge [Collewijn et al. 1988; Enright 1984; Gibaldi and Banks 2019; Zee et al. 1992]. These vergence biases are consistent with natural-disparity statistics. Consequently, the biases ensure that when the eyes land at the end of a saccade in the real world they will be fixating the most likely distance of the new target. This speeds up visual processing because it minimizes the likelihood of having to make another vergence movement to accurately fixate the new target.

For these reasons, it is very important that the horopter and oculomotor biases are compatible with the statistics of the natural environment. Otherwise, these biases would be counter-productive.

**Fig. 11.**
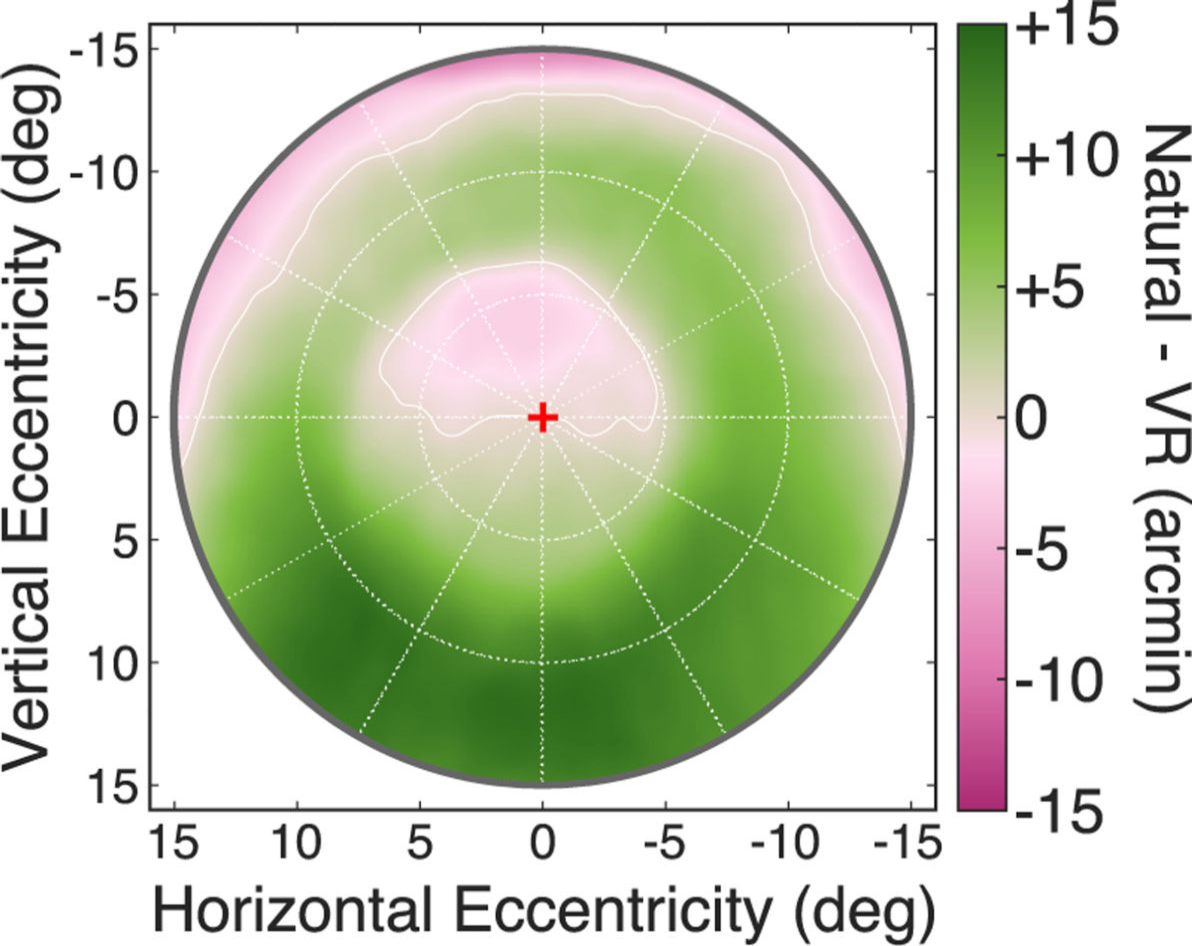
Differences between median disparity in VR and natural environments. The difference—natural minus VR—is plotted for all positions in the visual field. The color bar indicates disparity difference in minutes of arc. Green regions are where the natural disparities are more positive (crossed) than the VR disparities. Purple is where they are more negative (uncrossed).

Now consider the disparities in the VR-gaming environment. The upper left panel of Figure 10 shows median disparities in retinal coordinates across the visual field in that environment. The median disparities are qualitatively similar to those from the natural environment. The VR statistics exhibit a bottom-to-top change from positive to negative disparity (near to far) and the left-toright change from negative to zero and back to negative. But these changes are smaller and less systematic in the VR environment than in the natural. We highlight this in Figure 11, which plots the difference between the median disparities (natural–VR) for each position in the visual field. There is a prominent difference in the lower field where disparity is decidedly more positive in the natural than in the VR environment. Unlike the natural-environment data, the bottom-to-top change in the VR data is not large enough to match the horopter’s pitch. And the left-right change is not large enough to match the horopter’s horizontal curvature. We hypothesize that solving the binocular correspondence problem, obtaining fusion, achieving precise stereo vision, and making accurate vergence during saccadic eye movements are compromised in the VR-gaming environment.

**Fig. 12.**
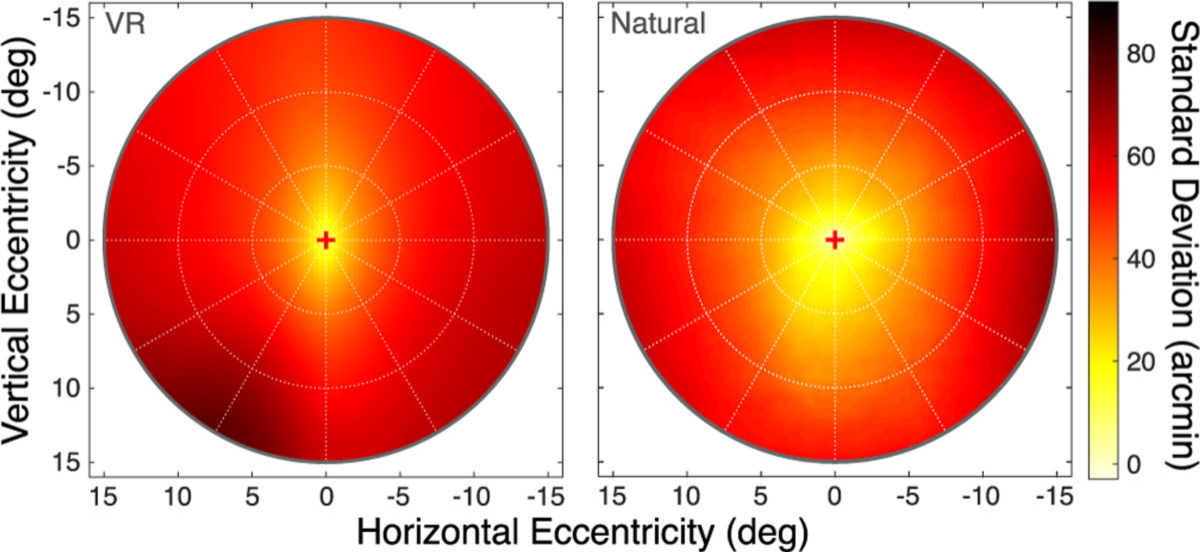
Standard deviation of horizontal disparity. The left panel shows the data from the VR environment and the right panel the data from the natural environment. Again, the VR data have been averaged across the four games and 10 subjects and the natural data have been averaged (with weighting) across six everyday tasks and four subjects.

**Fig. 13.**
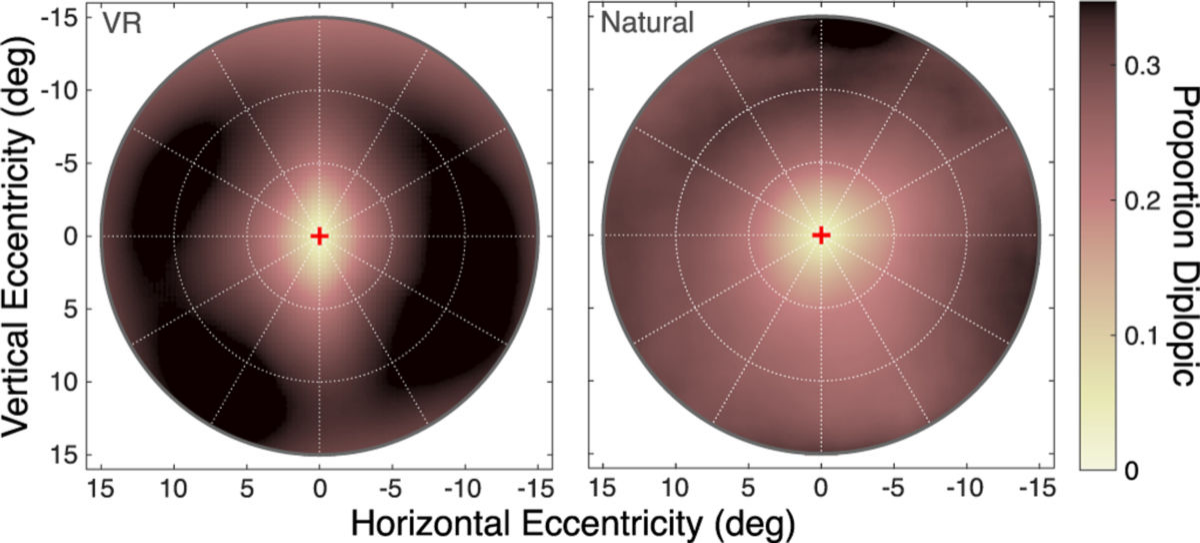
Proportion of disparities that would produce double vision in VR-gaming and natural environments. (Left) Proportion for the VR environment. (Right) Proportion for the natural environment.

We next examined the variability of disparity in the two environments (Figure 12). In the natural environment (right panel), the standard deviation increases roughly in proportion to eccentricity from a value close to 0° at the fovea to 60–80 arcmin at an eccentricity of 10°. This systematic change in disparity variation is reflected in the functional structure of the binocular visual system. The range of disparities that produce a fused image (i.e., not a double image) grows in proportion to retinal eccentricity [Hampton and Kertesz 1983; Ogle 1950]. The standard deviation in the VR environment increases more with eccentricity than in the natural environment, particularly in the left and right visual fields. We explored an implication of this finding by calculating from the disparity statistics the probability of experiencing double vision across the visual field. To do this, we modeled Panum’s fusion area (the range of fusable disparities) using data from previous psychophysical experiments [Ames et al. 1932; Ogle 1950]. We then collated data on the shape of the horopter [Cooper et al. 2011; Gibaldi and Banks 2019; Grove et al. 2001; Nakayama 1977; Schreiber et al. 2008]. We centered the range of fusable disparities on the horopter. We then created a smooth 3D surface that best fit the horopter data:

(1) $$D_{H}=-0.0485Y-0.0036X^{2}-0.0017Y^{2},$$

where X and Y are Helmholtz azimuths and elevations in degrees, and $D_{H}$ is the horizontal disparity of the surface, also in degrees. We used a similar method to model Panum’s fusion area [Ames et al. 1932; Hampton and Kertesz 1983; Ogle 1950]. The equation providing the best fit is

(2) $$D_{F}=D_{H}\pm \left(0.16+0.095|\epsilon |+|\epsilon {|}^{1.35}\right),$$

where ϵ is eccentricity of the visual direction in degrees: $\epsilon =\sqrt{X^{2}+Y^{2}}$. We then calculated for each field position the proportion of observed disparities that would fall outside of the fusable range. The results for the VR-gaming and natural environments are plotted in the left and right panels of Figure 13, respectively. Clearly, the proportion of disparities that could produce double vision is greater in the VR environment, particularly in the left and right fields.

We also observe that the spread of horizontal disparity in the natural environment is much greater than the spread of vertical disparity. Specifically, the aspect ratio of the joint distribution of horizontal and vertical disparity is ~20:1. This statistical property is manifest in the binocular visual system. For example, cortical neurons in primates have much more variation in their preferred horizontal disparity than in their preferred vertical disparity [Cumming 2002; Durand et al. 2007]. Furthermore, when presented stereoscopic stimuli in which the direction of disparity (e.g., horizontal, vertical, or oblique) is ambiguous, humans exhibit a strong bias to assume that the direction is horizontal [Rambold and Miles 2008; Van Ee and Schor 2000]. The spread of horizontal disparity relative to that of vertical disparity in the VR-gaming environment is ~16:1, which is quite similar to the natural ratio. Thus, this aspect of disparity in the virtual environment is consistent with natural statistics.

## 6 METHODS FOR USER EXPERIMENT

We designed an experiment in the HMD to test whether having scene content consistent with the statistics of the natural environment affects viewer comfort and performance. To our knowledge, this is the first such test for virtual environments.

### 6.1 Apparatus

The HMD and controllers were the same as in the fixations and disparities experiment.

### 6.2 Participants

Sixteen subjects participated. They were 20–61 years of age, had better than 20/32 visual acuity as measured by the Bailey-Lovie chart [Bailey and Lovie 1980], and stereothresholds of less than 30 arcsec on the Randot stereopsis test [Okuda et al. 1977]. They could all read the content presented in the HMD.

### 6.3 Procedure

The experiment was conducted in one session for each participant. Participants were shown black text on a white page and told to read it out loud. The text was from *Harry Potter and the Sorcerer’s Stone* [Rowling 1997]. Each trial had two presentation intervals with a 1 s inter-stimulus interval in between. There were two types of trials: Tilt (2/3 of the trials) and Magnitude (1/3). Participants were shown 30 trials in total. Viewing distance was 66 cm. At that distance, the slant of the vertical horopter is on average 16.6°. Its tilt is always 90° [Cooper et al. 2011; Ogle and Ellerbrock 1946].

**Fig. 14.**
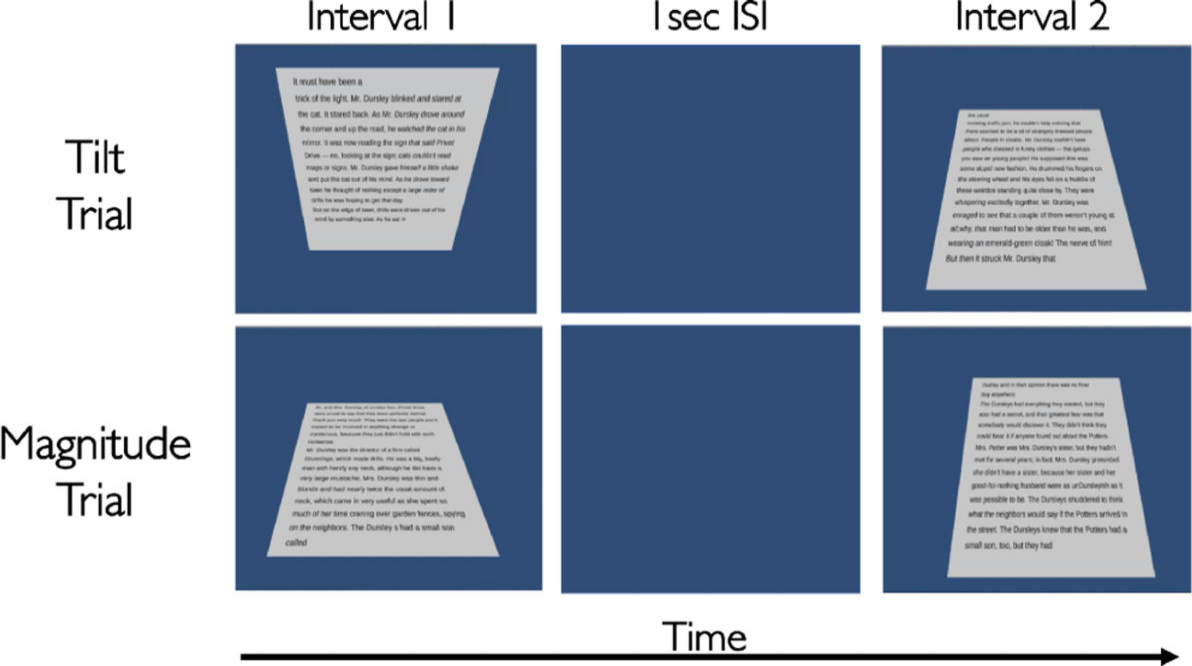
Examples of the two types of trials and the stimuli. The top row shows an example of a Tilt trial. The stimulus page is top forward (tilt = 270°) in the first interval and top back (90°) in the second. Both have a slant of 30°. The bottom row shows a Magnitude trial. The pages are both top back (tilt = 90°) and the slants are 40° in the first interval and 20° in the second.

For the Tilt trials, the stimulus page was slanted top back in one interval (tilt = 90°, consistent with the horopter ) and top forward in the other (tilt = 270°, inconsistent with the horopter), as shown in the upper row of Figure 14. The order of top-back and top-forward stimuli was randomized. Slant was the same in both intervals: 20°, 30°, 40°, or 50°. Participants were shown each slant five times for a total of 20 trials. The stimulus in each interval was presented until the participant had completed reading the page out loud. At the end of the two intervals, he/she indicated with a keypress which page was more comfortable to read. We also measured how long it took for the participant to read the page in each interval.

For the Magnitude trials, the stimulus pages were either both top back (tilt = 90°) or both top forward (tilt = 270°), as shown in the bottom row of Figure 14. The slants differed; they were random pairings of 20°, 30°, 40°, and 50° with the constraint that the two slants were different. Again, participants read out loud and indicated which of the two intervals was more comfortable to read. And again, we measured how long it took to read the page in each interval. Ten Magnitude trials were presented to each participant. Tilt and Magnitude were presented in the same session in random order.

## 7 RESULTS OF USER EXPERIMENT

**Fig. 15.**
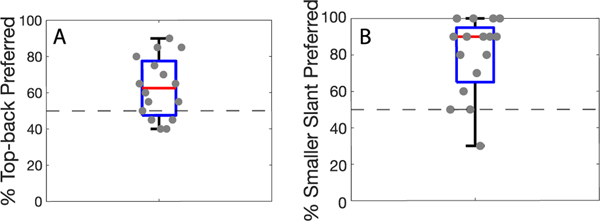
Results from the user experiment. (A) Tilt trials. Percentage of trials in which top-back slant was judged as more comfortable than top-forward. (B) Magnitude trials. Percentage of trials in which the smaller slant was judged as more comfortable. The dashed lines at 50% indicate no preference. Medians are represented by the red lines. The top and bottom of the blue boxes represent the 25th and 75th percentiles, respectively. Gray points are individual data points, shifted horizontally to aid visualization.

Figure 15(a) shows the results for the Tilt trials. It plots the percentage of trials in which the top-back slant was deemed more comfortable than the top-forward slant. The dashed line at 50% indicates no preference between the two. Higher values indicate greater preference for top-back. Participants preferred the top-back page significantly more often than the top-forward (one-sided *t*-test relative to 50%: *t*(15) = 3.06, *p* = 0.004). A one-way ANOVA across slants revealed no effect of slant on the preference for top-back (*F*(3) = 1.26, *p* = 0.29). In other words, participants consistently preferred top-back stimuli no matter what the slant was. This result is consistent with our expectation that stimuli that are more consistent with natural-scene statistics lead to more comfortable experiences.

Participants also read the top-back text slightly faster than the top-forward: 24.6 vs. 25.1 s/page. This difference was significant (*t*(14) = −2.08, *p* = 0.03), showing that performance is better with content that is consistent with the natural environment than with content that is not. There was no significant effect of slant on reading speed (*F*(3) = 1.38, *p* = 0.26) which shows that the improvement in performance with top-back slant was consistent across slants. We might have observed a larger difference between the top-back and top-forward stimuli if we had employed silent reading because out-loud reading is constrained by non-sensory, motor components while silent reading is not [Brysbaert 2019]. In other words, reading rate may have been constrained by a ceiling effect associated with speech production. We remind the reader, however, that we chose out-loud over silent reading to make sure that participants actually read the whole page.

Figure 15(b) shows the results for the Magnitude trials. It plots the percentage of trials in which smaller slants were deemed more comfortable than larger ones. Again, higher values indicate a preference for smaller slants and the dashed line indicates no preference. Participants significantly preferred the smaller slant (one-sided *t*-test against 50%: *t*(15) = 5.48, *p* < 0.0001).

The results of the user experiment show that stimuli that are consistent with natural statistics (and the horopter) are more comfortable to read and yield better reading performance than stimuli that are inconsistent with natural statistics. These are important results that we hope will influence HMD and video-game design.

## 8 DISCUSSION

We measured the statistics of fixations and disparities in the VR-gaming environment and compared them to those in the natural environment. We noted differences in the two environments that might affect visual comfort and performance. We showed experimentally that conforming to the statistics of the natural environment increases reading performance and user comfort. We now discuss further implications.

### 8.1 Field of View in HMDs vs Natural Viewing

We observed (Figure 5), as others have, that the direction of gaze is concentrated more straight ahead in HMDs than in natural viewing [Kollenberg et al. 2010; Pfeil et al. 2018; Sidenmark and Gellersen 2019; Sitzmann et al. 2018]. We hypothesize that this is due to: (1) the smaller field of view in HMDs, (2) how eye movements affect field of view in HMDs compared to natural viewing, and (3) how image quality affects fixation directions.

With respect to the first item, the horizontal and vertical fields of view in natural viewing are, respectively, ~200° and ~150°. The horizontal and vertical fields in HMDs are much smaller. In the Vive Pro Eye they are 94° (total field; 72° binocular) and 93°. Because of the limited field, HMD users must rotate their heads more frequently to see objects of potential interest than they have to in natural viewing.

With respect to the second item, eye movements affect the field of view differently in HMDs and natural viewing. In HMDs, the part of the virtual world an eye can see is fixed to the head because the display device is fixed to the head. As a consequence, making leftward and rightward eye movements does not expand the field seen by an eye; they simply shift the visible field across the retina. This is more complicated in natural viewing. The nasal field limit is imposed by the nose and bony orbit. The temporal limit is imposed by the *ora serrata*: the position in the retina where photoreceptors terminate. Thus, the nasal limit is fixed to the head and the temporal limit to the retina. As a result, leftward and rightward eye movements expand the field seen by an eye. If one makes a leftward (or rightward) eye movement in natural viewing, the visible field expands leftward (or rightward). We hypothesize, therefore, that viewers make larger eye movements in natural viewing than in HMDs because they can expand the effective visible field by so doing.

With respect to the third item, HMDs with Fresnel optics have poorer image quality in the peripheral parts of the screen than in the center. As a result, viewers might avoid directing their foveal line of sight into regions of lower quality, choosing instead to move their heads to bring eccentric objects into the center of the screen.

### 8.2 Screen Displacement

**Fig. 16.**
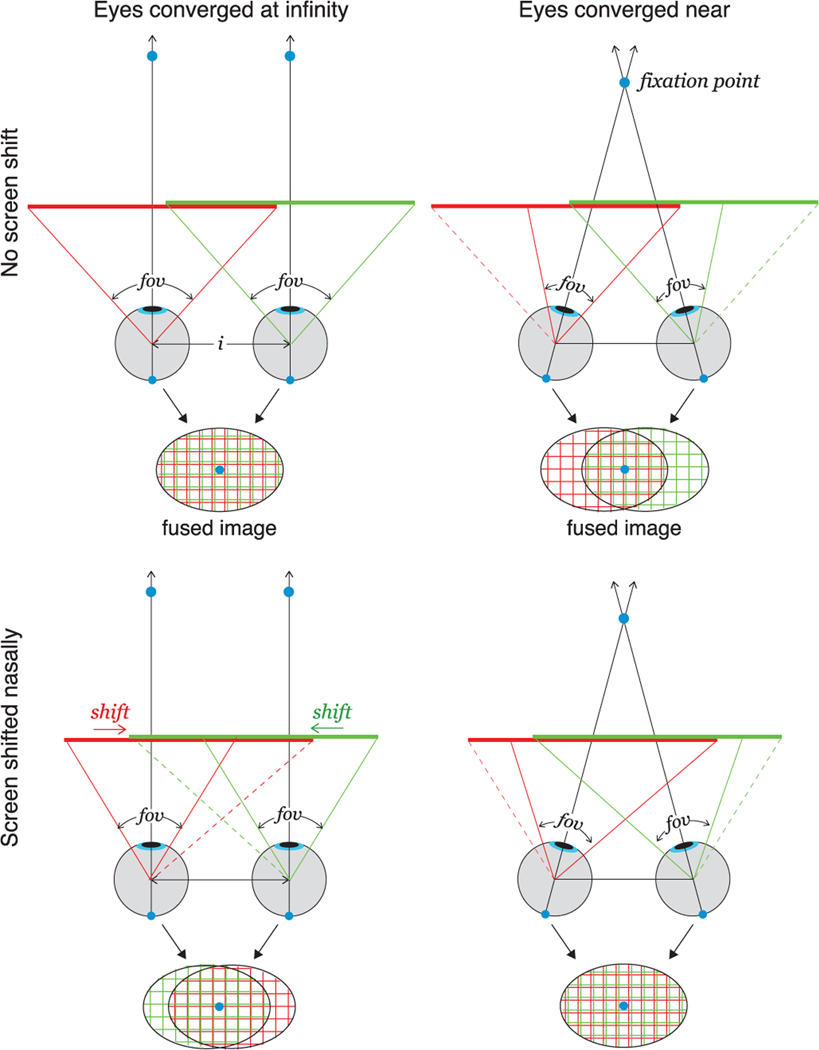
Geometry of binocular field of view. The display screens are represented by the thick red and green lines. The foveas are indicated by blue dots at the back of the eyes. The binocular field of view is represented by *fov*. In the upper panels, the screens are symmetric about the lines of sight for eyes are that are not converged. In the lower panels, the screens are shifted nasally. The eyes are converged at infinity and at a near distance in the left and right panels, respectively. The binocularly fused images are indicated by ellipses below the eyes. The red grid represents the part of the screen seen by the left eye and the green grid the part seen by the right eye. The foveas are indicated again by blue dots. In the upper left panel, the screen parts seen by the two eyes are superimposed, so the binocular field is the same width as the monocular fields. In the upper right panel, the fused images are displaced temporally because the eyes are converged. The binocular field is the part where the red and green grids are superimposed. It is narrower than in the left panel. In the lower right panel, the eyes are converged so the nasal shifts of the screens create a wider binocular field of view.

The screens in most HMDs have a wider temporal field than nasal field, which increases the total field of view (the regions seen by one or the other eye). But this temporal bias decreases the binocular field of view (the regions that are imaged on corresponding regions in the two eyes). It is interesting to consider these fields of view along with the statistics of gaze (Figures 5 and 7). Figure 16 helps explain how we examined this. It shows how screen size and positioning and fixation distance affect the binocular field of view. The left and right panels show, respectively, the situations with the eyes fixating at infinity (parallel lines of sight) and at a near distance. The upper and lower halves of the figure show, respectively, the situations when the screens are symmetric about the line of sight (i.e., eyes fixating ahead at infinity) and when the screens are shifted nasally. The width of the field seen by both eyes on corresponding retinal regions is indicated by *fov*. With symmetric screens (upper panels) the binocular field of view is widest (and identical to the two monocular fields) when the eyes are converged at infinity. But when the eyes converge, the lines of sight intersect the screens at successively more nasal points, and the binocular field narrows. The ellipses at the bottom of the upper panels represent the fused binocular images. The red grid is the part of the field seen by the left eye and the green grid is the part seen by the right eye. The binocular field of view is the intersection of the two monocular fields. The total field of view is the union of the monocular fields. With nasally shifted screens (lower panels), the binocular field is wider when the eyes are converged.

**Fig. 17.**
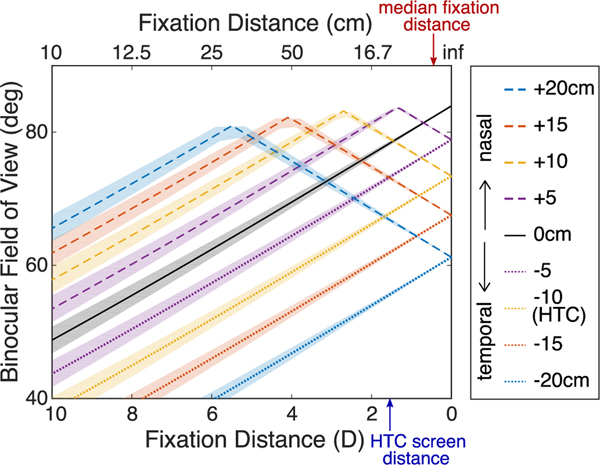
Binocular field of view, fixation distance, and screen position. The width of the binocular field is plotted as a function of the distance to which the eyes are converged and the horizontal shifts of the two display screens. Fixation distance is plotted in diopters on the lower axis and centimeters on the upper. Curves of different colors represent field size for different screen shifts. Black is no displacement (screens symmetric with lines of sight with forward gaze and eyes converged at infinity). Dashed lines represent displacements of both screens nasalward. Dotted lines represent displacement temporalward. An inter-ocular distance of 6.33 cm is assumed; shaded areas represent ±1 standard deviation of inter-ocular distance [Dodgson 2004]. The yellow dotted line represents field size for the HTC Vive Pro Eye which has a temporalward shift of ~10 cm. The blue arrow indicates screen distance in the Vive Pro Eye and the red arrow the median fixation distance in the VR-gaming statistics.

Figure 17 shows how the width of the binocular field of view depends on fixation distance and whether the screens are shifted nasally or temporally relative to straight ahead. The screens in the simulation are both 117 cm wide at an optical distance of 65 cm (as in the Vive Pro Eye). The widest binocular field for symmetric screens (i.e., shift = 0 cm) is 84° and is achieved when the eyes are converged at infinity. The Vive Pro Eye has temporal shifts of ~10 cm so the binocular field (yellow dotted line) is only 72° in that device when the eyes are fixated at infinity. Temporal shifts decrease the binocular field and nasal shifts increase it, especially at nearer fixation distances. Our data on the statistics of fixations in VR video games (Figure 6) revealed a median fixation distance of ~150 cm (0.7D), which is indicated by the red arrow. For this fixation distance, symmetric screens (shift = 0 cm) yield a binocular field of ~81° while asymmetric screens like the Vive Pro Eye (shift = −10 cm) yield a binocular field of just 70°. A wider binocular field of view is achieved for the median fixation distance by shifting the screens nasally by 5 cm. Furthermore, the binocular field is wider for nearly all fixation distances with 5 cm nasal shifts than with no shift or temporal shifts. This expansion of the binocular field is maintained when subjects make leftward or rightward movements while keeping the same fixation distance. Thus, HMDs would be more effective in presenting stereo information for likely fixation distances if the screens were shifted nasally. Of course, expanding the binocular field of view (the part seen by both eyes) is associated with shrinking the total field of view (the part seen by the left eye or right eye, or both), so the display designer must evaluate the tradeoff between binocular and total field of view.

### 8.3 Adverse Effects Due to Deviations from Natural Environment

There are a variety of negative consequences for presenting environments that do not conform to the regularities we observed for the natural environment.

1. Binocular fusion is determined by the 3D location of an object relative to the horopter and Panum’s fusion area. As we said earlier (Section 5.3), the horopter is pitched top back. This means that surfaces that are also slanted top back are more likely to create a fused impression than surfaces that are pitched top forward. A compelling example of this is the *Venetian-blind effect* [Piggins 1978; Tyler 1980]. (A demonstration is provided in Supplementary Figure S3.) A pattern of vertical stripes on a planar surface is viewed binocularly. The surface is then rotated about the horizontal axis. When the slant is top forward, the pattern is not properly fused and a series of steps in depth is seen: a Venetian blind. When the slant is top back, the pattern can be properly fused and the illusory depth steps are not seen. Thus, surfaces that are consistent with the top-back pitch of the horopter are more fusable than surfaces that are inconsistent.
2. Ergonomic researchers advise computer users to pitch desktop displays slightly top back to minimize viewing discomfort [Ankrum et al. 1995; Grandjean et al. 1983]. The top-back pitch is consistent with the pitch of the horopter and with natural-disparity statistics (Section 5.3). Environments that do not conform to the horopter produce more discomfort.
3. Panum’s fusional area is centered on the horopter and increases in proportion to retinal eccentricity [Hampton and Kertesz 1983; Ogle 1950], which means that the objects in the parafovea and periphery can have larger disparities before they produce a double (non-fused) percept. In the natural environment, the range of disparities is proportional to retinal eccentricity (Figure 12), so the probability of experiencing non-fused, double imagery is roughly constant across the visual field (Figure 13). Our observations for the VR-gaming environment show that the range of disparities in that environment is not proportional to eccentricity (Figure 12). In particular, the range in the left and right visual fields is quite large, so double imagery should be experienced more often in that environment than in the real world (Figure 13). Furthermore, video games do not generally incorporate depth-of-field blur as it is experienced in the real world. The lack of depth-of-field blur increases the likelihood of diplopia because Panum’s fusion area is smaller for sharp than for blurred objects [Schor et al. 1984].
4. Oculomotor behavior should be consistent with natural statistics. When people make upward saccades, they tend to diverge the eyes. This is the same but to a lesser degree for leftward and rightward saccades. When people make downward saccades, they tend to converge [Collewijn et al. 1988; Enright 1984; Gibaldi and Banks 2019]. These biases are useful because they ensure that the eyes at the end of a saccade are most likely to be aligned with the new fixation target. Because the statistics in the VR-gaming environment are not congruent with those in the natural environment, the relationship between saccades in different directions and the appropriate vergence is disrupted and should cause delays in the acquisition of new targets in the VR environment.
5. The vergence-accommodation conflict causes discomfort, poorer performance, and distortions of 3D perception [Koulieris et al. 2019; Kramida 2015; Lambooij et al. 2009; Urvoy et al. 2013]. We found that such conflicts are common in the VR-gaming environment because players tend to fixate consistently farther in the virtual scene than the distance of the screen (Figure 6). Thus, it is commonplace for significant vergence-accommodation conflicts to occur in that environment.

## 9 CONCLUSION

In summary, we found that fixation directions and distances are more restricted in VR-gaming environments than in the natural environment. And that fixation distances are considerably farther in virtual environments. We used our data to calculate the screen distance and positioning that would, respectively, minimize discomfort and maximize the binocular field of view. We also found that the patterns of retinal disparity encountered in VR-gaming and natural environments are quite different from one another. The pattern is more variable in the virtual environment and does not exhibit the top-back pitch to the same degree as observed in the natural environment. Our user experiment showed that stimuli that are consistent with natural statistics (and the horopter) are more comfortable to read and yield better reading performance than stimuli that are inconsistent with natural statistics.

Our investigation was limited to one type of headset and just four video games. It would be useful to expand this analysis to other headsets and other types of VR experience. It would be interesting as well to measure head movements as people experience virtual and natural environments in order to compare the combined eye and head movements made in these environments. We showed how the binocular field of view can be widened for common fixations, but this comes with a narrowing of the total field of view. It would be useful to determine what the best tradeoff is between expanding the binocular field versus expanding the total field.

## Supplementary Material

Video frames

Supplementary video

## Footnotes

1 During the course of this research, new methods enabled such capability [Hartmann et al. 2019; Thoravi Kumaravel et al. 2020], but we did not have access to the methods in time to use in our study.
