# Supplementary material for: The Statistics of Eye Movements and Binocular Disparities during VR Gaming: Implications for Headset Design
Source: ACM Trans Graph. Author manuscript; Available in PMC 2023 Apr 27. (PMC10139447; doi:10.1145/3549529)
Supplement: Video frames [file NIHMS1877680-supplement-Video_frames.pdf]

Supplementary Material for: The Statistics of Eye Movements and Binocular Disparities During VR Gaming: Implications for Headset Design

AVI M. AIZENMAN, GEORGE A. KOULIERIS, AGOSTINO GIBALDI, VIBHOR SEHGAL, DENNIS M. LEVI, and MARTIN S. BANKS, University of California, Berkeley, Durham University

COMPUTING RETINAL DISPARITY

In order to obtain a proper estimate of retinal binocular disparity one must to know the 3D structure of the visual scene and the eye posture with respect to such scene.

3D Scene. For the Natural Environment setup, first we estimated the distortion coefficients, the intrinsic and extrinsic parameters of the stereoscopic scene camera using a standard stereo calibration procedure [Bouguet 2004; Zhang 2000]. The stereoscopic images are first rectified using the distortion coefficients, and a semi-global matching algorithm [Hirschmüller 2007] is next used to compute the stereoscopic disparity. The intrinsic and extrinsic parameters of the stereo rig are then used to convert the disparity map into the 3D scene geometry, i.e., the depth buffer of the scene. For the VR environment, this information is directly provided by the rendering engine. The depth buffer is then transformed into the 3D point cloud corresponding to the visual scene, using projective geometry.

Binocular Eye Posture. The pose of a camera can be defined by six degrees of freedom, three for the position and three of the orientation in the 3D space. Considering a human observer, the position of each eye is defined by the baseline, since the cyclopean point corresponds to the origin of the reference frame O, and each eye is displaced of half of the baseline in opposite directions along the x-axis. The orientation of the eyes is defined by the binocular extension of Listing's Law [Somani et al. 1998; Tweed 1997; Van Run and Van den Berg 1993], which specifies the amount of the torsional angle with respect to the binocular gaze direction.

© 2022 Copyright held by the owner/author(s).
0730-0301/2022/09-ART7
https://doi.org/10.1145/3549529

Retinal Disparity.

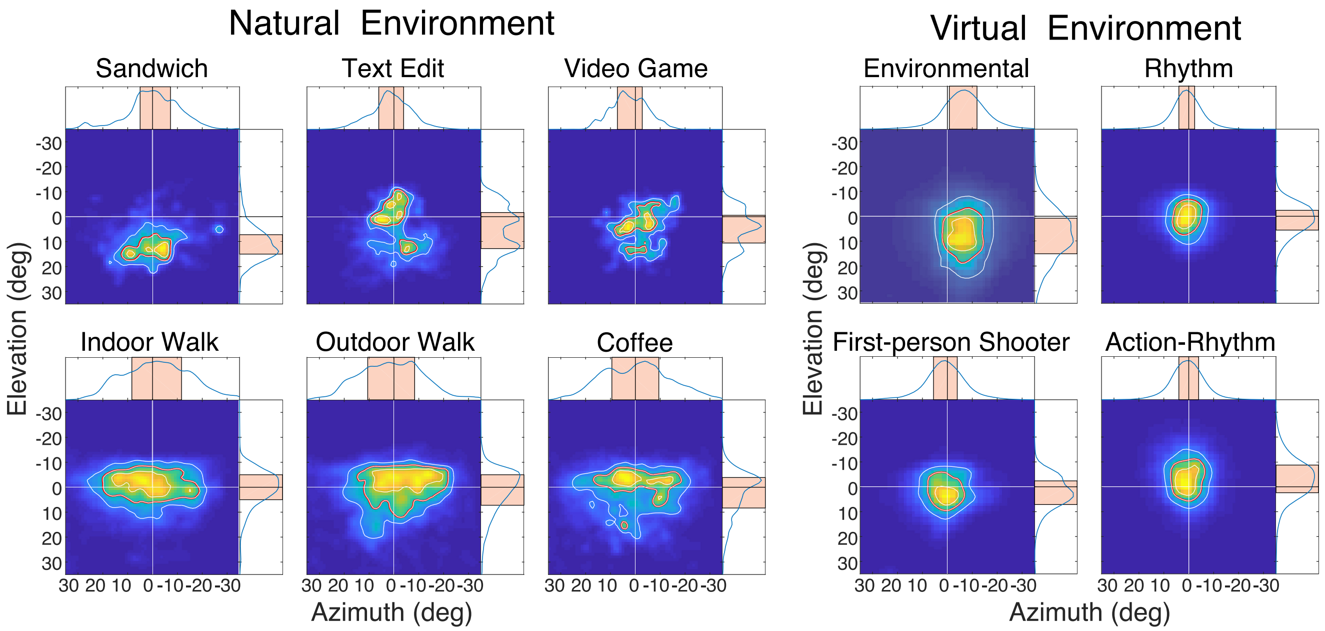

Fig. S1. Probability of fixation direction for each natural task and video game. Horizontal gaze direction on the horizontal axes and vertical direction on the vertical axes. The red contours represent 50% of fixations. The white contours are 25th and 75th percentiles. Marginal probabilities are shown on the right and top. The red areas represent 50% of fixations.

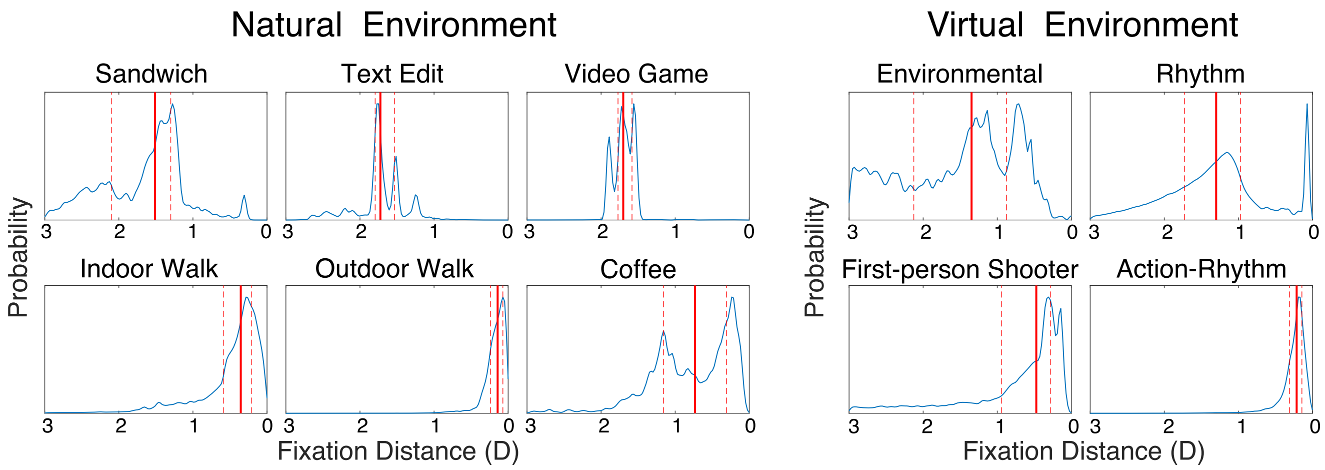

Fig. S2. Probability of fixation distances for each natural task and video game. Fixation distance is plotted in diopters: Near distances on the left and far on the right. Median fixation distances are represented by the red lines and 25th and 75th percentiles by the red dashed lines.

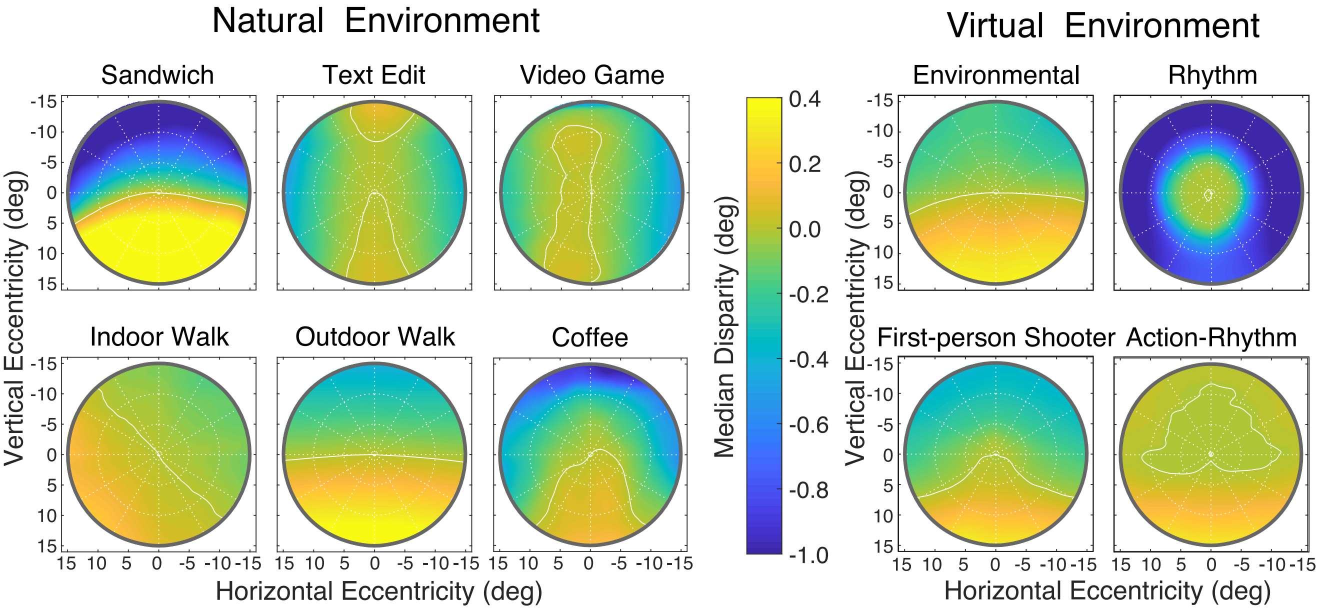

Fig. S3. Median disparity across the visual field for each natural task and video game. Median horizontal disparity is plotted in each panel. Fovea is in the middle. The white curves show where median disparity is zero. The data for the six natural tasks are shown on the left and the data for the four video games are shown on the right.

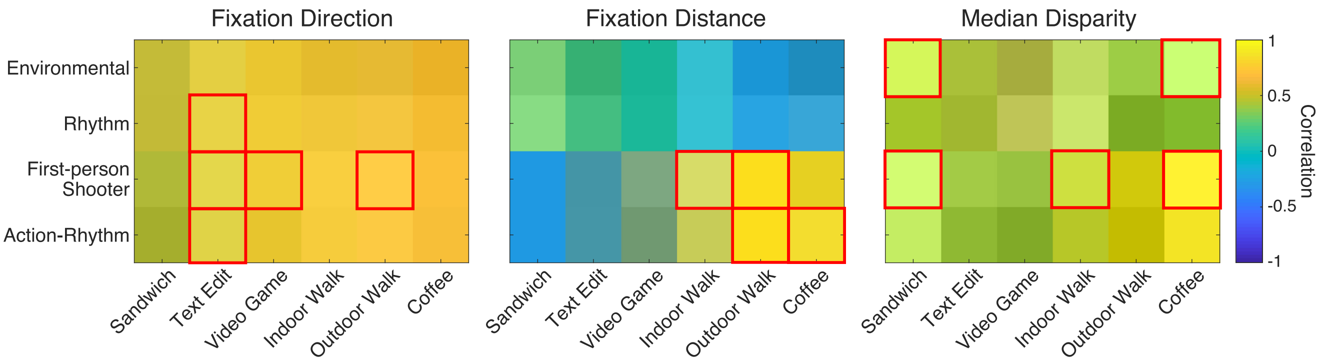

Fig. S4. Correlations between data from the natural and VR gaming environments. Pearson's correlation was computed between the two environments for fixation direction (left), fixation distance (center), and median disparity (right). Each square represents the correlation between the data from a natural task (horizontal axis) and the data from a VR game (vertical axis). Red squares indicate correlations >0.75.

REFERENCES

Jean-Yves Bouguet. 2004. Camera calibration toolbox for MATLAB. http://www.vision.caltech.edu/bouguetj/calib_doc/index.html. (2004).

Heiko Hirschmüller. 2007. Stereo processing by semiglobal matching and mutual information. *IEEE Transactions on Pattern Analysis & Machine Intelligence* 30, 2 (2007), 328–341.

Rizwan A. B. Somani, Joseph F. X. Desouza, Doug Tweed, and Tutis Vilis. 1998. Visual test of Listing's law during vergence. *Vision Research* 38, 6 (1998), 911–923.

Douglas Tweed. 1997. Visual-motor optimization in binocular control. *Vision Research* 37, 14 (1997), 1939–1951.

L. J. Van Run and Albert V. Van den Berg. 1993. Binocular eye orientation during fixations: Listing's law extended to include eye vergence. *Vision Research* 33, 5–6 (1993), 691–708.

Zhengyou Zhang. 2000. A flexible new technique for camera calibration. *IEEE Transactions on Pattern Analysis & Machine Intelligence* 22, 11 (2000), 1330–1334.
